# Mixed-Effects Modeling Framework for Amsterdam and Copenhagen for Outdoor NO_2_ Concentrations Using Measurements Sampled with Google Street View Cars

**DOI:** 10.1021/acs.est.1c05806

**Published:** 2022-03-09

**Authors:** Jules Kerckhoffs, Jibran Khan, Gerard Hoek, Zhendong Yuan, Thomas Ellermann, Ole Hertel, Matthias Ketzel, Steen Solvang Jensen, Kees Meliefste, Roel Vermeulen

**Affiliations:** † Institute for Risk Assessment Sciences, Utrecht University, 3584 CK Utrecht, Netherlands; ‡ Department of Environmental Science, Aarhus University, DK-4000 Roskilde, Denmark; § Danish Big Data Centre for Environment and Health (BERTHA), Aarhus University, DK-4000 Roskilde, Denmark; ∥ Department of Bioscience, Aarhus University, DK-4000 Roskilde, Denmark; ⊥ Global Centre for Clean Air Research (GCARE), University of Surrey, GU2 7XH Guildford, U.K.; # Julius Centre for Health Sciences and Primary Care, University Medical Centre, University of Utrecht, 3584 CK Utrecht, The Netherlands

**Keywords:** Google Street View, NO_2_ measurements, LUR, mixed-effect model, hyperlocal variation

## Abstract

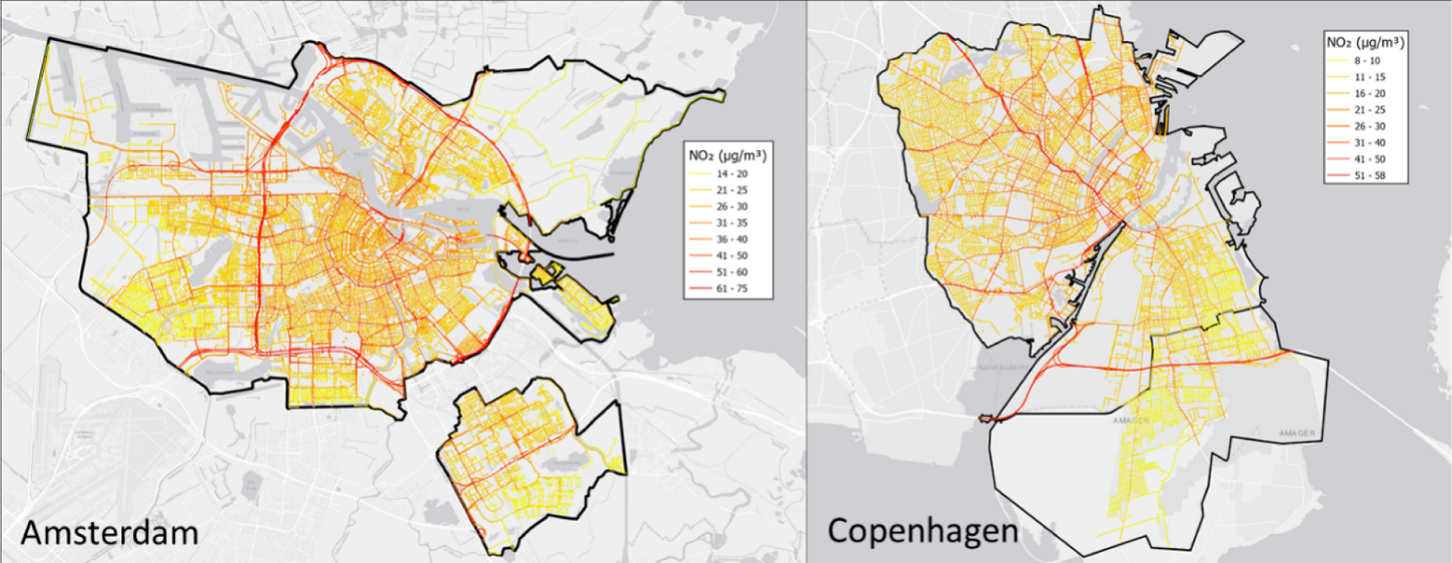

High-resolution air quality (AQ) maps based on street-by-street measurements have become possible through large-scale mobile measurement campaigns. Such campaigns have produced data-only maps and have been used to produce empirical models [i.e., land use regression (LUR) models]. Assuming that all road segments are measured, we developed a mixed model framework that predicts concentrations by an LUR model, while allowing road segments to deviate from the LUR prediction based on between-segment variation as a random effect. We used Google Street View cars, equipped with high-quality AQ instruments, and measured the concentration of NO_2_ on every street in Amsterdam (*n* = 46.664) and Copenhagen (*n* = 28.499) on average seven times over the course of 9 and 16 months, respectively. We compared the data-only mapping, LUR, and mixed model estimates with measurements from passive samplers (*n* = 82) and predictions from dispersion models in the same time window as mobile monitoring. In Amsterdam, mixed model estimates correlated *r*_s_ (Spearman correlation) = 0.85 with external measurements, whereas the data-only approach and LUR model estimates correlated *r*_s_ = 0.74 and 0.75, respectively. Mixed model estimates also correlated higher *r*_s_ = 0.65 with the deterministic model predictions compared to the data-only (*r*_s_ = 0.50) and LUR model (*r*_s_ = 0.61). In Copenhagen, mixed model estimates correlated *r*_s_ = 0.51 with external model predictions compared to *r*_s_ = 0.45 and *r*_s_ = 0.50 for data-only and LUR model, respectively. Correlation increased for 97 locations (*r*_s_ = 0.65) with more detailed traffic information. This means that the mixed model approach is able to combine the strength of data-only mapping (to show hyperlocal variation) and LUR models by shrinking uncertain concentrations toward the model output.

## Abstract 2

The mixed-effects modeling framework is a robust and scalable approach for mobile measurements for mapping air pollution at a very fine spatial resolution.

## 1 Introduction

Most air pollutants exhibit small-scale spatial variation that cannot be captured by traditional routine monitoring networks. Exposure assessment of air pollution has, therefore, been revolutionized via mobile monitoring platforms during the past decade.^1−16^ With advancements in air monitoring instrumentation, such as higher time resolution and greater portability, mobile monitoring platforms can directly measure spatial gradients and peaks in urban air pollution. Li et al.^17^ showed that quantifying spatial variation of NO_2_ within urban areas with high fidelity (<4 μg/m^3^ NO_2_) is not likely attainable unless the sampling network is dense, having more than one or two sensors per km.^2^ Whereas mobile sampling is great in measuring the local variation in concentration levels, a fundamental limitation is that such measurements only consist of a limited number of seconds per street segment.^17^ To reduce this problem, most mobile monitoring designs used land-use regression (LUR) models to develop concentration maps. Alternatively, when a significant number of repeated measurements are available, these could be used to create measurement-only concentration maps.^3,16,18^ Both approaches have strengths and limitations.

Regarding data-only mapping, Robinson et al.^18^ considered 15 days as the minimum threshold of the daily visits required to produce representative long-term air pollution concentrations. This value is based on the work conducted by Apte et al.,^16^ who designed a mobile sampling campaign using Google Street View (GSV) cars to measure air pollution levels on all streets in Oakland, USA. In Apte’s study, each street segment was measured around 50 times to develop a high-resolution measurement-only air pollution map of the city.

However, measuring each street segment in a region of interest requires a significant amount of time, which might not be feasible for many locations, particularly in bigger cities (e.g., >100 km^2^). Therefore, many researchers have combined mobile monitoring with empirical LUR models to produce air pollution maps.^3,4,15^ To compare data-only maps with LUR models, Messier et al.^3^ measured all streets in Oakland at least 50 times and assumed that driving 50 times on different days generates “robust” long-term average concentrations. The authors then reduced the number of measuring days and compared data-only maps with the LUR models. They found that data-only mapping performed poorly with a few repeated drives, for example, one to two drives, but obtained *R*^2^ values better than the LUR approach within four to eight repeated drive days per road segment. A limitation of LUR models is however the loss of the very high spatial resolution as LUR models tend to “smooth” concentration levels over a wider terrain.^19^

Therefore, in this paper, we propose a mixed modeling framework that combines the strengths of both data-only mapping and LUR models. This framework can deal with limited mobile monitoring data per street segment and “preserve” the high spatial resolution as much as possible. This method uses all measurements on all street segments to develop a LUR model but allows individual measurements to influence the output based on the between and within-segment variation. All measurements and models were averaged over street segments as the goal is to create a spatial map with long-term exposure predictions. We used mobile NO_2_ measurements collected with GSV cars in Amsterdam and Copenhagen to test and evaluate this framework. We compared data-only NO_2_ concentrations, LUR, and mixed model estimates with measurements from passive samplers and routine monitoring networks. We additionally compared with deterministic model predictions.

## 2 Materials and Methods

### 2.1 Study Sites

**Figure 1:**
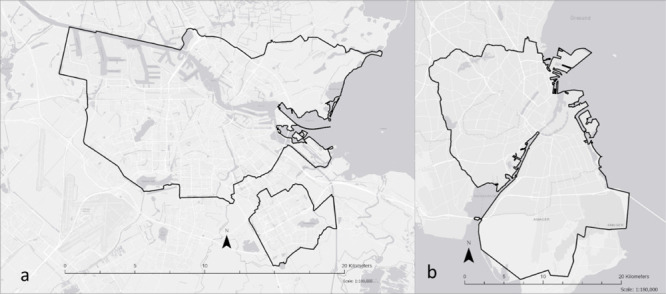
Study sites: (a) City of Amsterdam and (b) Copenhagen metropolitan area containing three municipalities, Copenhagen, Frederiksberg, and Tårnby. The bold black lines show the border of the study sites. Background maps ESRI.

Amsterdam (hereafter, AMS) is the capital and the largest city of the Netherlands (see Figure 1a). AMS is the most populous city and has one of the densest road networks in the Netherlands. The city center has a mix of residential and commercial mid- and high-rise buildings and is bound by major interstate highways (Figure 1a). Amsterdam airport is located south-west of the city. AMS is flat (with surrounding flat land) and has an oceanic climate, significantly affected by its proximity to the North Sea to the west, with prevailing westerly winds.

The second study site consists of three municipalities, Copenhagen, Frederiksberg, and Tårnby, in the Copenhagen metropolitan area with a large commuter belt surrounding Copenhagen (see Figure 1b). Copenhagen (hereafter, CPH) is the largest and most populous city in Denmark and the Danish capital located on the eastern shore of the island of Zealand and Amager. The central part of CPH is relatively flat. The urban area stretches up to pprox.. 292 km^2^. CPH is interspersed with residential and commercial blocks containing low-, mid-, and high-rise buildings including major highways. The Copenhagen airport is in the south of the metropolitan area (Figure 1b).

### 2.2 Data Collection

Three GSV cars were equipped with 1 Hz nitrogen dioxide (NO_2_) monitors (CAPS, Aerodyne Research Inc, USA) and used to measure NO_2_ concentrations on each street segment in AMS and CPH. The instrument directly measures NO_2_ concentrations based on optical scattering and absorption. The geographical location of the car was recorded via a Global Positioning Unit (GPS; G-Star IV, GlobalSat, Taiwan), which was linked to the NO_2_ monitor in the GSV car using date and time. We used two GSV cars to monitor concentrations in AMS from 25 May 2019 to 15 March 2020. The third car was used to monitor NO_2_ concentrations in CPH from 15 October 2018 to 15 March 2020. Both measurement campaigns were stopped on 15 March 2020 due to COVID-19 lockdown restrictions. Measurements were collected between 08:00 and 22:00 on weekdays, with most measurements between 10:00 and 16:00. During data collection, the GSV cars measured in different parts of AMS and CPH as much as possible to reduce the spatial–temporal autocorrelation. NO_2_ concentrations higher than 500 μg/m^3^ and lower than 0 μg/m^3^ were removed from the data set as they are unrealistic and clear outliers. The final data set consisted of 5.9 million and 5.1 million 1 Hz measurements of NO_2_ in AMS and CPH, respectively. All processing steps, including subsequent model developments and analyses, are done in R software, version 4.0.4.

### 2.3 Data Processing and Aggregation

As street segments were measured at different times of the day and week, we applied a temporal correction to the measured data using nearby reference stations (one per city), explained in detail in the Supporting Information. In brief, the difference between the overall average concentration and the average of specific time windows at the reference station was used to correct all mobile measurements in corresponding time windows. The reference station measured concentrations for the full time period (all days of the week and day and night) of the mobile monitoring campaign, so corrected measurements can be used to reflect long-term concentrations.

All measurements were assigned to the nearest street. The assigned values were then averaged over 50 m street segments per individual driving day (hereafter, drive-pass). Subsequently, we computed a mean of all drive-passes to get a single “mean of means” for all street segments. On average, each street segment consisted of 8 [interquartile range (IQR): 3–10] seconds per drive-pass and seven unique drive-passes, with some streets having multiple hours of data. There were 46,664 and 28,499 total street segments in the road network of AMS and CPH, respectively. Data of all drive-passes were used to develop the mixed-effects model for AMS and CPH. The “mean of means” data were used for data-only mapping and as inputs to develop LUR models for AMS and CPH. LUR models were developed by a supervised linear forward stepwise regression model. The criteria used in the development of the LUR models and coefficients for each city can be found in the Supporting Information.

### 2.4 Mixed Model Development

We developed a mixed modeling framework, also known as a linear “mixed-effects” model. The term comes from the coexistence of both fixed and random effects. The fixed effects are obtained from the standard coefficients of the LUR model. As all road segments are measured, we can use the measurements on all street segments as a random effect (cluster-specific effect). This allows the inclusion of cluster-specific effects while borrowing strength/stability from the fixed effects. This borrowing is stronger when data are closer to the average effect or for clusters that have less data. This way, the measured hyperlocal variation is preserved while uncertain low or high concentrations are shrunken toward the LUR model output. The mixed-effect model can be expressed as
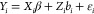
where *Y* is the mixed model prediction. The second part starts with the fixed effect where β is a (*p*, 1) vector of fixed effects attached to a matrix (*X*) of (*n*_*i*_, *p*) covariates. Then, the random effects are added where *b*_*i*_ is a (*q*, 1) vector of random effects attached to a matrix (*Z*) of (*n*_*i*_, *p*) covariates. The regression parameters, β (the fixed effects parameters), are the same for all individual drive-passes. If the vector of random effects *b*_*i*_ has mean zero, the mixed model estimates are fully based on the fixed effects (LUR model). Mixed model results were then averaged per street segment, similar to the average of the data-only approach and the LUR model.

### 2.5 Comparison with External Monitoring and Modeling

To evaluate the mixed model performance for AMS and CPH, we compared data-only measurements, LUR, and mixed model estimates with monitoring networks and deterministic model predictions. Hereafter, the data-only, LUR, and mixed model estimates, altogether, are referred to as Amsterdam Air View (AAV) and Copenhagen Air View (CAV) data. All comparison data sets are listed in Table 1, and their details are provided below.

**Table 1:** Overview of GSV Data and Comparison Data Sets in Amsterdam and Copenhagen

city	data	number of sites	year	name
AMS	Amsterdam Air View data (data-only, LUR, mixed model)	46,664	2019–2020	AAV
	Palmes tubes measurements^20^	82	2019–2020^a^	Palmes
	model predictions by the National Institute for Public Health and the Environment^21^	7004	2019	NSL
	Dutch National Air Quality Monitoring Programme	7	2019	LML
CPH	Copenhagen Air View data (data-only, LUR, mixed model)	28,499	2018–2020	CAV
	AirGIS model predictions (2019)^22,23^	58,234	2019	LPDV
	AirGIS model predictions along streets	97	2019	CPH-97
	Danish National Air Quality Monitoring Programme^24^	3	2018–2020^a^	NOVANA

a Matches exact time window of GSV measurements. AMS: Amsterdam; CPH: Copenhagen.

For AMS, the AAV data were compared with measurements from a passive sampler network using Palmes tubes at facades of buildings, which are maintained by the Municipal Health Service (GGD).^20^ The Palmes tubes data consisted of repeated 4-weekly measurements throughout the whole year, covering all AMS and its surroundings. A total of 82 sites were within 20 m of the AAV measurements and had measured data available in the exact time window of the AAV campaign.

The AAV data were also compared with the model predictions from the Dutch National Collaboration Programme on Air Quality [In Dutch: “Nationaal Samenwerkingsprogramma Luchtkwaliteit” (NSL)].^21^ Model predictions from this framework are calculated for each major road at 100 m intervals on both sides, approximately 10 m from the roadside. We compared AAV data with the nearest NSL prediction within 20 m (*n* = 7004). In addition, we also compared AAV NO_2_ concentrations, Palmes, and NSL, where all three data sources were available (*n* = 47, overlapping sites).

To assess the “absolute levels” of NO_2_ concentrations across the city, we compared mixed model estimates with annual average NO_2_ concentrations collected by the Dutch National Air Quality Monitoring Programme (LML).

For CPH, the CAV data were compared with three air quality datasets. The first comparison dataset was based on recently updated Air Quality at Your Street address-level NO_2_ concentrations, annual average, 2019 (hereafter, LPDV).^23^ LPDV is a high-resolution public map of air quality for each address location in Denmark. The air pollution levels were estimated using the Danish multiscale dispersion modeling system (DEHM-UBM-AirGIS), a standard toolkit to calculate pollution levels at any address location in Denmark. The modeled concentrations are representative of close to the building façade. The details of the DEHM-UBM-AirGIS system as well as its detailed inputs are provided in the study by Khan et al., 2019. CAV data were compared to the nearest LPDV point within 20 m (*n* = 58,234).

The second comparison dataset was based on high-quality DEHM-UBM-AirGIS^22^ predictions of NO_2_ concentrations and point locations along 97 busy streets in Copenhagen. Air pollution (e.g., NO_2_) is usually calculated for these streets as part of the Danish National Monitoring and Assessment Programme for the Aquatic and Terrestrial Environment (NOVANA). Again, the nearest neighbor analysis, as described above, was performed to compare NO_2_ estimates. This comparison dataset will be referred to as CPH-97. This dataset is based on more detailed and validated traffic data than LPDV as traffic data originate from the traffic monitoring program of the Municipality of Copenhagen.

The third comparison dataset (NO_2_, 2019 annual averages) was acquired from two traffic monitoring stations and two background stations. The monitoring stations are part of the Danish Air Quality Monitoring Network in four major cities of Denmark; see Ellermann et al.^24^ for more details. These data (hereafter, NOVANA) are used to assess the “absolute levels” of NO_2_ concentrations across the city.

## 3 Results

In the results section, we split the analyses by city and combine interpretations in the Discussion section.

### 3.1 Amsterdam

The LUR model based on measurements for AMS is shown in Supporting Information Table A2. The model mainly includes variables that describe local traffic intensity. Furthermore, the model includes a large-scale population density variable and the area of ports within a 1000 and 5000 m buffer. The model was able to explain the average concentrations per street segment moderately well (*R*^2^ value 0.49).

**Figure 2:**
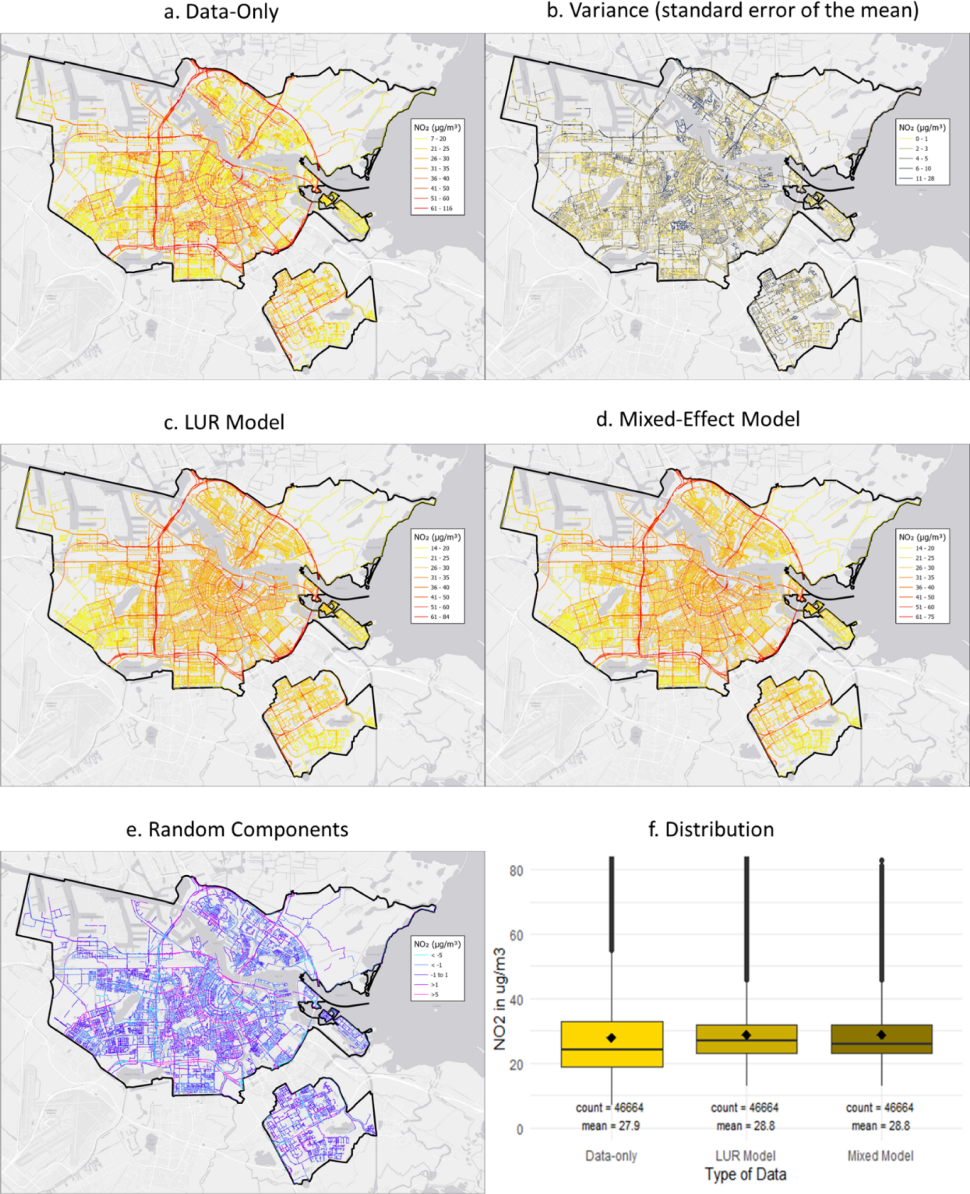
Maps of measurements, predictions, and variance in Amsterdam. (a) Data-only map, (b) standard error of the mean, (c) LUR model (fixed effects), (d) mixed-effect model, (e) random components, and (f) distribution of NO_2_ measurements and predictions. Note: Boxes represent the IQR; the horizontal line is the median; vertical lines extend to IQR times 1.5 (limited to data); dots are individual outliers; the black squared dot is the mean. Full size maps in the Supporting Information (Figure A1).

Figure 2 shows the data-only map (a), followed by the variance map with the standard error of the mean (b). This map indicates that multiple street segments have a large absolute and/or relative uncertainty. Figure 2c shows the predictions by the LUR model, which is much more smoothed than the data-only map. The mixed model prediction map (Figure 2d) is more smoothed than the data-only map but incorporates the variance of the data-only map in the random effect. This leads to more preservation of the local effects. Figure 2e shows, via the random effects (i.e., the difference between the mixed model and the LUR model), that the overall variance is comparable to the variance of the data-only map. In Figure 2f, we show the distribution of the measurements and model predictions by the LUR model and the mixed-effect model. High-resolution NO_2_ maps are available in the Supporting Information (Figure A1) and the mixed model estimates via Google’s Environmental Insights Explorer (https://insights.sustainability.google/labs/airquality). For all datasets, the concentrations are higher along the highways/major roads and vary generally smoothly along less busy roads. The same variation of pollution was also observed in the city center of AMS. Figure 2f shows that the variation in data-only NO_2_ is higher than the LUR and mixed model estimates.

In Table 2, we present the summary statistics and Spearman correlation coefficients of all datasets (including external datasets) with matching locations. Measurements by the GSV car (and subsequent mixed model output) are on average higher than measurements and predictions by the Palmes tubes and NSL. Concentration distributions for the external datasets are given in Supporting Information Figure A2.

**Table 2:** Summary Statistics, Correlation, and Bias Scores for all Comparisons in AMS^a^

	summary statistics					correlation and bias			
	min	Q1	med	Q3	max	*r*_s_	RMSE	mean bias	mean relative bias (%)
Comparison to NSL Predictions, *n* = 7004									
NSL	12	23	26	28	41				
data-only	7	23	29	37	116	0.50	11.35	5.2	20
LUR model	16	27	31	34	84	0.61	6.76	5.1	20
mixed model	15	27	30	35	69	0.65	7.47	5.5	21
Comparison to Palmes Measurements, *n* = 82									
Palmes	17	23	28	32	44				
data-only	13	23	34	42	58	0.74	10.23	6.3	23
LUR model	20	28	32	35	52	0.75	6.49	4.5	16
mixed model	17	28	34	38	57	0.85	7.67	6.1	22
Comparison to Palmes Measurements with Overlapping NSL Sites, *n* = 47 (Major Roads Only)									
Palmes	19	26	31	33	41				
NSL	23	29	33	35	40	0.54	4.84	2.1	7
data-only	16	32	36	43	54	0.74	9.95	6.5	22
LUR model	24	32	35	37	50	0.45	6.93	4.7	16
mixed model	23	33	35	41	51	0.72	8.04	6.4	21

a Summary statistics, RMSE, and mean bias in μg/m^3^. Min = minimum, Q1 = the 25th percentile, med = median or the 50th percentile, Q3 = the 75th percentile, max = maximum, *r*_s_ = Spearman’s rank correlation, RMSE = root-mean square error, mean bias calculated as mean [(ref – test), mean relative bias calculated as mean bias/ref] where: ref = NSL, Palmes, and test = AAV data.

Correlations between all data sets were moderately high, with the highest correlation between the mixed model and Palmes tubes (*r*_s_ = 0.85). For data-only and LUR model predictions, correlations were 0.74 and 0.75, respectively. Furthermore, at Palmes sites with overlapping NSL predictions (*n* = 47), the mixed model explained measured concentrations at major roads modestly better than the national dispersion model predictions. Since NSL only makes predictions on major roads, the total variation in concentrations drops, resulting in overall lower correlation scores compared to the full set of monitoring locations. It also shows that a LUR model has more difficulties predicting concentration levels within that higher category, whereas the data-only approach is able to achieve a similar performance compared to the complete validation set. For the entire NSL dataset, we also found slightly higher correlations for the mixed model output than data-only and LUR model outputs. Supporting Information Figure A4 shows the scatterplots and Bland Altman plots for all comparisons.

Of note, AAV data and mixed model predictions were on average 6.3 and 6.1 3 μg/m^3^ higher than the measurements from the Palmes tubes (Table 2). The main reason for this difference is the fact that AAV data are measured and predicted on the road, while Palmes measurements were performed on the façade of buildings and expected to be lower due to dilution from road to building façade. Comparing the absolute concentration levels from the mixed model with mean concentrations from the seven routine measurement stations (LML) in AMS over 2019, we found a difference of 3 μg/m^3^, which is about 10%. In Supporting Information Figure A3, we show a bar chart for all seven LML sites. We found no apparent differences for sites close to traffic and sites in an urban background environment. Both data sets do not exactly overlap as the GSV was conducted from May 2019 till February 2020, and the routine measurements are the annual averages of 2019.

### 3.2 Copenhagen

The developed LUR model based on measurements in CPH is shown in Supporting Information Table B2, and like the AMS LUR model, it mainly includes variables that describe the local traffic intensity. However, the CPH LUR model also includes traffic intensity variables with bigger buffers and the average building height within 100 m. As noted in Section 2.5, the estimated/average building height was only available for CPH. The *R*^2^ value of the model was slightly higher than that of the AMS LUR model, that is, *R*^2^ = 0.54.

**Figure 3:**
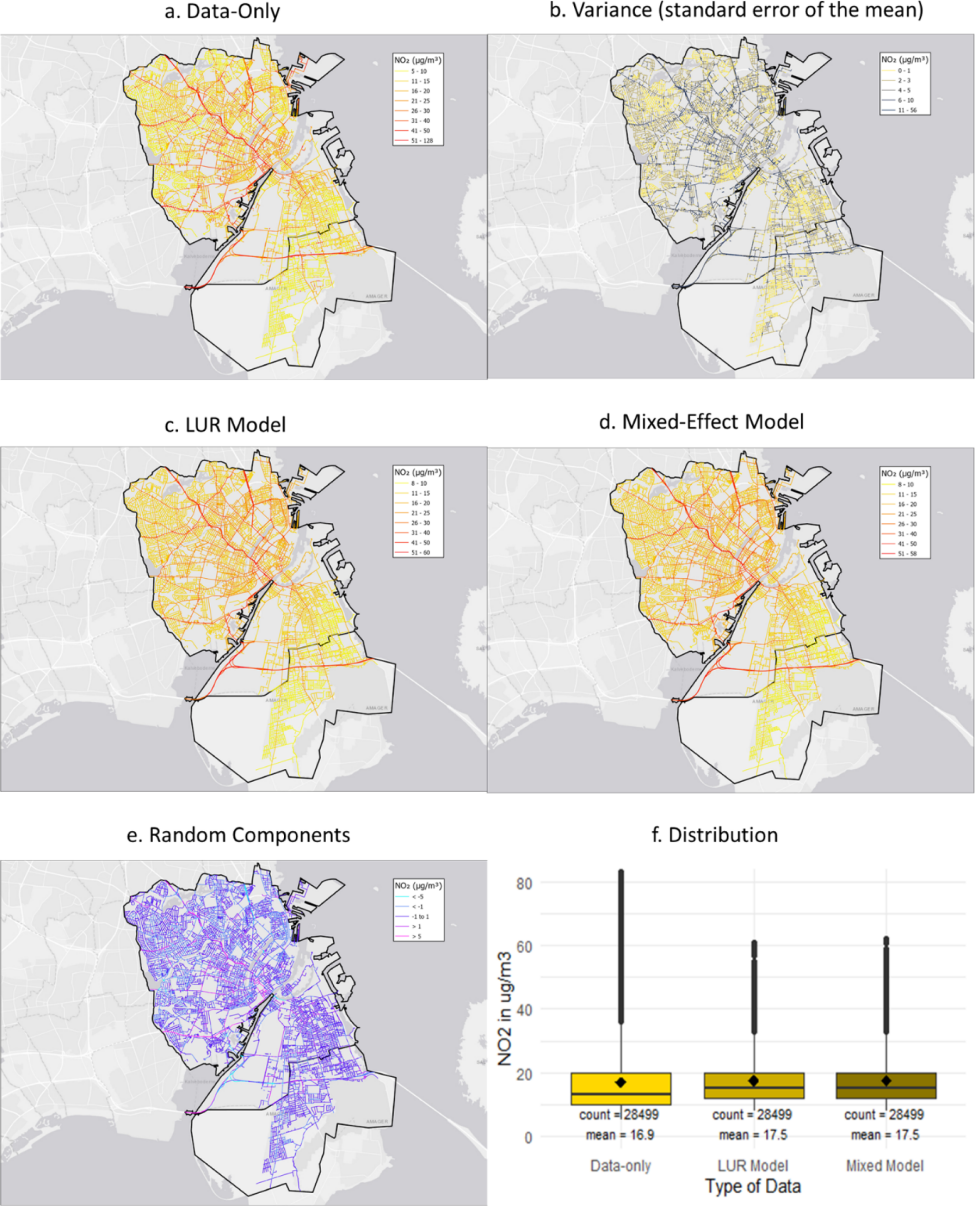
Maps of measurements, predictions, and variance in Copenhagen. (a) Data-only map, (b) standard error of the mean, (c) LUR model (fixed effects), (d) mixed-effect model, (e) random components, and (f) distribution of NO_2_ measurements and predictions. Note: Boxes represent the IQR; the horizontal line is the median; vertical lines extend to IQR times 1.5 (limited to data); dots are individual outliers; the black squared dot is the mean. Full size maps in the Supporting Information (Figure B1).

Figure 3 shows the data-only map of Copenhagen (a), followed by the variance map with the standard error of the mean (b). Like AMS, there are differences in absolute and/or relative uncertainties between street segments. Figure 3c shows the predictions by the LUR model, which is much more smoothed than the data-only map. The mixed model prediction map (Figure 3d) is more smoothed than the data-only map but incorporates the variance of the data-only map in the random effect. This leads to increased hyperlocal variability of concentrations. The random effects are shown in Figure 3e, showing that the overall variance is comparable to the variance of the data-only map. In Figure 3f, we show the distribution of the measurements and model predictions by the LUR model and mixed-effect model. High-resolution maps are available in the Supporting Information (Figure B1) and the mixed model estimates also via Google’s Environmental Insights Explorer (https://insights.sustainability.google/labs/airquality). Like Amsterdam, the concentrations are higher along the highways/major roads and vary generally smoothly along less busy roads, with variation in data-only NO_2_ being slightly higher than the other datasets.

In Table 3, we present the distribution of measurements and models and Spearman’s correlation coefficients for CPH. Correlations with CAV data were moderately high for the CPH-97 data set but decreased when CAV data were compared with the LPDV data. CPH-97 has higher concentrations than LPDV data because CPH-97 only includes near-traffic locations. Mixed model estimates agreed better with the dispersion model approaches (i.e., LPDV, CPH-97) than the data-only and LUR models. Table 3 also shows that the mixed model is able to lean to a LUR model when this generates better predictions (for LPDV) and uses more data-only measurements when they are more robust (for CPH-97). Supporting Information Figure B4 shows the scatterplots and Bland Altman plots for all comparisons.

**Table 3:** Summary Statistics, Correlation, and Bias Scores for all Comparisons in CPH^a^

	summary statistics					correlation and bias			
	min	Q1	med	Q3	max	*r*_s_	RMSE	mean bias	mean relative bias
Comparison to LPDV Model Predictions (*N* = 58,234)									
LPDV	11	14	15	18	47				
data-only	5	10	13	17	128	0.45	7.18	–1.87	–11
LUR model	8	12	15	18	50	0.50	4.06	–1.08	–6
mixed model	8	12	15	18	53	0.51	4.15	–1.14	–7
Comparison to CPH-97 Model Predictions (*N* = 97)									
CPH-97	17	22	25	28	39				
data-only	12	19	25	31	53	0.67	7.75	0.81	3
LUR model	16	24	27	30	50	0.55	5.16	1.78	7
mixed model	16	23	27	30	52	0.65	5.94	2.04	8

a Summary statistics, RMSE and mean bias in μg/m^3^. min = minimum, Q1 = the 25th percentile, med = median or the 50th percentile, Q3 = the 75th percentile, max = maximum, *r*_s_ = Spearman’s rank correlation, RMSE = root-mean square error, mean bias calculated as mean [(ref – test), mean relative bias calculated as mean bias/ref] where: ref = LPDV, CPH-97, and test = CAV data.

In CPH, we did not find significant higher measurements and predictions by the CAV car compared to the LPDV data (Table 3), though we found similar differences between CAV mixed model estimates and four stationary sites (Figure B3). Differences were between 10 and 20% in terms of absolute values, except for H.C. Andersen’s Boulevard, where concentrations differ by about 30%.

## 4 Discussion

In one of the largest mobile monitoring campaigns to date, we have shown that mobile monitoring can be used to develop accurate air pollution maps. The applied mixed model approach uses the advantages of a data-only and an empirical (LUR) model approach, outperforming the two individual approaches when compared to external measurements and different national dispersion models. Since all road segments are measured, the mixed models use the hyperlocal variation that can be picked up by a data-only approach while borrowing the stability from the LUR model estimates. This way, the measured hyperlocal variation is preserved, while uncertain low or high concentrations are shrunken toward the LUR model output.

### 4.1 Mobile Monitoring

Studies based on mobile monitoring usually face one out of two problems: the high variance (noise) in mobile measurements for specific locations (road segments) or loss of hyperlocal spatial variation by the creation of a LUR model. Figures 2b and 3b show that the variance (standard error of the mean) differs significantly between streets and neighborhoods. For example, 15% of the street segments in both cities have a standard error of the mean bigger than 5 μg/m^3^. While for some streets, four to eight repeats will be enough to characterize long-term concentration, some streets remain uncertain. Interpretation of hyperlocal effects is therefore very difficult.

Only a few mobile monitoring campaigns have been able to measure such a significant amount of repeated measurements on street segments in a specific area, and there was no need to build a LUR model in order to create an air pollution concentration map.^3,7,16^ While Messier et al.^3^ found that 4–8 repeats were sufficient to create a data-only map for black carbon and nitrogen oxide (NO) better, or at par with a LUR model, Miller et al.^7^ sampled each street segment (*n* = approx. 10,500) in Harris County, Texas 15–44 times and Apte et al.^16^ needed 1 year to sample each street segment (*n* = approx. 21,000) in different parts of Oakland at least 30 times. It takes a lot of time and effort to create such rich data (>15 drives). For AMS and CPH with 46,664 and 28,499 street segments, respectively, it would take much more time or cars to achieve, let alone scaling up to bigger and more areas.

Nevertheless, data-only mapping in AMS correlated highly with external measurements (*r*_s_ = 0.74; Table 2). On the other hand, data-only mapping in CPH correlated poorly with the national model predictions (*r*_s_ = 0.45; Table 3).

### 4.2 LUR Model Development

In previous work,^4^ we showed that LUR models based on only two to three visits per street segment could predict external long-term measurements with moderately high accuracy. In Messier et al.,^3^ the authors found that even with 2 drive days per road segment, the *R*^2^ value, that is 0.52, was within 15% of models developed on 45+ drive-passes. Hatzopoulou et al.^15^ decreased the number of road segments from 611 to 100 in steps of 50, and *R*^2^ values remained stable up until 200 road segments. Even LUR models based on 100 segments predicted on average 73% of the variation (opposed to 74% for the entire dataset), albeit with a wider confidence interval (55–85% opposed to 70–78% for the entire dataset). Two other studies in Canada also found that increasing the visits (or total measurement time) quickly stabilizes LUR model predictions based on mobile measurements.^25,26^

In this study, *R*^2^ values for the LUR models in AMS and CPH were also moderately high (0.49 and 0.54; see Tables A1 and B1). Of note, *R*^2^ values depend not only on the number of drive-passes or total time spent on a road segment but also on the urban topography of a city and the type of input data available. European cities tend to be more spatially diverse than North American cities, making it harder for LUR models to explain the variability of air pollution.^27^

Nonetheless, predictions made with LUR models correlated high with external measurements (*r*_s_ = 0.75) and moderately, that is, *r*_s_ = 0.50–0.61, with model predictions (Tables 2 and 3). This is similar to correlations with data-only mapping. Because of the smoothing of LUR models, RMSE and mean bias values are lower than data-only mapping and the mixed model approach, especially in Amsterdam. This mainly happens at the higher end of the concentration scale; see Figures A4 and B4. This relates to LUR models typically less able to capture small-scale variation compared to data-only mapping. This is mainly due to the fact our LUR models incorporated traffic intensity but not features like the composition of traffic and speed. Other local features like street configuration and small industrial sources are also missing. The balance between data-only and LUR-model maps depends on how extensive and detailed predictor variables are available. More and better predictors likely increase the performance of LUR models, especially predictor data that can explain the very local variation of air pollution.

### 4.3 Mixed Model Development

By using a mixed modeling framework, we were able to take advantage of both measured concentrations per road segment and LUR modeling at the same time. LUR models are generally more stable but not so well at detecting local features. In Table 2, we show that the mixed modeling estimates correlated higher with external measurements (*r*_s_ = 0.85) compared to the data-only (*r*_s_ = 0.74) and LUR model output (*r*_s_ = 0.75). Mixed model estimates also correlated higher with external model predictions compared to the data-only and LUR model output (Tables 2 and 3). Spearman correlations were 0.65, 0.51, and 0.65, on average 0.1 higher than data-only mapping and LUR model estimates.

A mixed model approach in air pollution research is not new. Several studies used this method to assess spatial and temporal variations of air pollution at the same time.^28−31^ For these studies, the main goal was to create a model that can predict concentrations at other locations or at other time points. In our mixed model framework, we only used spatial land use information to create a long-term average map and do not need to predict concentrations at other locations or time points. The mixed-effect model was specifically used to bring in the hyperlocal variation in concentrations that is missed by a typical LUR model. Figures 2e and 3e show the difference between the LUR model and the mixed model. In other words, it shows the influence of the data-only mapping (random components). On about 10% of the street segments in CPH and 20% in AMS, there is difference of at least 3 μg/m^3^ between the LUR model and the mixed-effect model. The variance that is lost by the LUR model, compared to data-only map, is brought back by the random components of the mixed model.

### 4.4 Bias

For most comparisons, we found higher NO_2_ values for the data-only mapping, LUR, and mixed model method compared to all other external measurements and predictions, except the LPDV data. Several studies already reported that mobile monitoring studies create higher output values because these measurements are done in the middle of the road, while all external measurements and predictions are sampled on the side of the road or façade of buildings.

In previous studies to UFP (ultrafine particles) and BC (black carbon), we showed that predictions made by models based on mobile monitoring are about 20–30% higher than external home-outdoor stationary measurements.^4,32^ For NO_2_, the number seems to be slightly less, probably because NO_2_ is slightly less heterogeneously dispersed compared to UFP and BC due to photochemical reactions between NO and ozone-forming NO_2_, where NO emissions from the road are dispersed to the building façade. Experiments in real-world data also found steeper gradients for UFP and BC compared to NO_2_.^33−35^ In Tables 2 and 3, we show that NO_2_ predictions made by the mixed model output are about 15–20% higher than the external measurements and predictions. This is also shown in the Bland–Altman plots in the Supporting Information, where a larger bias is observed with higher concentration levels in all comparisons. Also, compared to official monitoring stations in AMS and CPH, the difference is about 15–30% (Figures A3 and B3). The same on-road/off-road ratio was found in a study by Richmond-Bryant et al.^36^ in Las Vegas. They found that NO_2_ concentrations declined from on-road to 10 m from the road by a median of 16% (and 21% for a 20 m distance).

This gradient of NO_2_ concentrations in the vicinity of roads (on-road/off-road ratio) depends on the wind direction and urban topography, making the exact ratio for each road segment individually hard to predict. The most practical solution would be to reduce mobile monitoring output by 20% for all road segments to approach residential exposure. Alternatively, the mixed model predictions could be combined with a dispersion model. Either by using mixed model predictions as line source in a dispersion model or by integrating both models in data fusion steps.

### 4.5 Strengths and Limitations

One of the strengths in this study is the fact that we were able to use external long-term measurements in the same time period as the mobile monitoring to validate our model predictions.^37^ Next, we were able to compare our model predictions with model predictions used by official national environmental agencies. Predictions in these models are made with dispersion models, meaning they are constructed very different than our empirical models. Differences between models can therefore not be interpreted as one being better than the other but rather that both models offer different features contributing to exposure estimates.

The biggest limitation of the measurement setup used in this work is the amount of time, energy, and significant initial investment it takes to collect such enormous amounts of data. In the study by Apte et al.,^16^ they estimated that it would take around 400 mobile monitoring vehicles to create a data-only map (>20 drives) for all streets in the largest 25 US cities, though the number of vehicles could be reduced if data are combined with LUR models. Within a mixed modeling framework this could easily be implemented, though it would need a huge effort in order to sample street segments in a large area (bigger than one or a few cities). A few drives are needed to develop a LUR model, while adding more and more drives increases the accuracy of data-only mapping. Hence, when more and more data are collected, actual measurements could explain more and more local variations. This makes the mixed model approach a very scalable solution to other cities as well. As the mixed model balances the input that is most accurate (data-only or LUR model estimates), there is no minimum number of drive-days to create a stable concentration map. This also means that the mixed model is able to predict concentration levels on street segments without measurements as they could be based on the LUR model output (with the limitations associated with LUR models in regard to smoothing of concentrations).

To keep the hyperlocal variation in air pollution maps, measurements on every street in question will always be needed. This could, for example, be achieved by putting measurement devices on municipal utility vehicles. Hasenfratz et al.,^10^ for example, collected over 50 million measurements of UFP over a 2 year period using mobile sensor nodes installed on top of public transport vehicles in the city of Zurich, Switzerland. While this effort did not cover the entire city, it contained enough data to develop a LUR model in a short amount of time. Coverage could be further increased when sensors or monitors are installed on utility vehicles that cover large parts of the city (e.g., municipality vehicles and delivery trucks). A mixed model approach will therefore always be at least as good as a LUR model as it takes the LUR model as the baseline and adds additional information based on the measurements.

## Supporting Information Available

Text on the temporal correction and LUR model development, definition of GIS predictors, LUR models, distribution of measurements and predictions, comparison between data-only, mixed model estimates, and external datasets, scatterplots and Bland–Altman plots for all comparisons with external datasets, and high-resolution version of all maps in the main text (PDF)

## List of Abbreviations and Acronyms

AQ: air quality
GSV: Google Street View
AMS: Amsterdam
AAV: Amsterdam Air View
CPH: Copenhagen
CAV: Copenhagen Air View
CPH-97: AirGIS model predictions along 97 busy streets in Copenhagen
LUR: land-use regression
LML: Dutch Air Quality Monitoring Programme
LPDV: Air Quality at Your Street Project 2019
NSL: Nationaal Samenwerkingsprogramma Luchtkwaliteit (Dutch National Collaboration Programme on Air Quality)
NOVANA: the National Monitoring and Assessment Programme for the Aquatic and Terrestrial Environment

## References

[ref1] Larson T.; Henderson S. B.; Brauer M. Mobile Monitoring of Particle Light Absorption Coefficient in an Urban Area as a Basis for Land Use Regression. Environ. Sci. Technol. 2009, 43, 4672–4678. 10.1021/es803068e. 19673250

[ref2] Patton A. P.; Collins C.; Naumova E. N.; Zamore W.; Brugge D.; Durant J. L. An Hourly Regression Model for Ultrafine Particles in a Near-Highway Urban Area. Environ. Sci. Technol. 2014, 48, 3272–3280. 10.1021/es404838k. 24559198 PMC4347899

[ref3] Messier K. P.; Chambliss S. E.; Gani S.; Alvarez R.; Brauer M.; Choi J. J.; Hamburg S. P.; Kerckhoffs J.; Lafranchi B.; Lunden M. M.; Marshall J. D.; Portier C. J.; Roy A.; Szpiro A. A.; Vermeulen R. C. H.; Apte J. S. Mapping Air Pollution with Google Street View Cars: Efficient Approaches with Mobile Monitoring and Land Use Regression. Environ. Sci. Technol. 2018, 52, 12563–12572. 10.1021/acs.est.8b03395. 30354135

[ref4] Kerckhoffs J.; Hoek G.; Vlaanderen J.; van Nunen E.; Messier K.; Brunekreef B.; Gulliver J.; Vermeulen R. Robustness of Intra Urban Land-Use Regression Models for Ultrafine Particles and Black Carbon Based on Mobile Monitoring. Environ. Res. 2017, 159, 500–508. 10.1016/j.envres.2017.08.040. 28866382

[ref5] Kerckhoffs J.; Hoek G.; Gehring U.; Vermeulen R. Modelling Nationwide Spatial Variation of Ultrafine Particles Based on Mobile Monitoring. Environ. Int. 2021, 154, 106569 10.1016/j.envint.2021.106569. 33866060

[ref6] Chambliss S. E.; Preble C. V.; Caubel J. J.; Cados T.; Messier K. P.; Alvarez R. A.; LaFranchi B.; Lunden M.; Marshall J. D.; Szpiro A. A.; Kirchstetter T. W.; Apte J. S.; Apte J. S. Comparison of Mobile and Fixed-Site Black Carbon Measurements for High-Resolution Urban Pollution Mapping. Environ. Sci. Technol. 2020, 54, 7848–7857. 10.1021/acs.est.0c01409. 32525662

[ref7] Miller D. J.; Actkinson B.; Padilla L.; Griffin R. J.; Moore K.; Lewis P. G. T.; Gardner-Frolick R.; Craft E.; Portier C. J.; Hamburg S. P.; Alvarez R. A. Characterizing Elevated Urban Air Pollutant Spatial Patterns with Mobile Monitoring in Houston, Texas. Environ. Sci. Technol. 2020, 54, 2133–2142. 10.1021/acs.est.9b05523. 31995368

[ref8] Qi M.; Hankey S. Using Street View Imagery to Predict Street-Level Particulate Air Pollution. Environ. Sci. Technol. 2021, 55, 2695–2704. 10.1021/acs.est.0c05572. 33539080

[ref9] Hankey S.; Marshall J. D. Land Use Regression Models of On-Road Particulate Air Pollution (Particle Number, Black Carbon, PM2.5, Particle Size) Using Mobile Monitoring. Environ. Sci. Technol. 2015, 49, 9194–9202. 10.1021/acs.est.5b01209. 26134458

[ref10] Hasenfratz D.; Saukh O.; Walser C.; Hueglin C.; Fierz M.; Arn T.; Beutel J.; Thiele L. Deriving High-Resolution Urban Air Pollution Maps Using Mobile Sensor Nodes. Pervasive Mob. Comput. 2015, 16, 268–285. 10.1016/j.pmcj.2014.11.008.

[ref11] Sabaliauskas K.; Jeong C.-H.; Yao X.; Reali C.; Sun T.; Evans G. J. Development of a Land-Use Regression Model for Ultrafine Particles in Toronto, Canada. Atmos. Environ. 2015, 110, 84–92. 10.1016/j.atmosenv.2015.02.018.

[ref12] Van den Bossche J.; Peters J.; Verwaeren J.; Botteldooren D.; Theunis J.; De Baets B. Mobile Monitoring for Mapping Spatial Variation in Urban Air Quality: Development and Validation of a Methodology Based on an Extensive Dataset. Atmos. Environ. 2015, 105, 148–161. 10.1016/j.atmosenv.2015.01.017.

[ref13] Farrell W.; Weichenthal S.; Goldberg M.; Valois M.-F.; Shekarrizfard M.; Hatzopoulou M. Near Roadway Air Pollution across a Spatially Extensive Road and Cycling Network. Environ. Pollut. 2016, 212, 498–507. 10.1016/j.envpol.2016.02.041. 26967536

[ref14] Weichenthal S.; Ryswyk K. V.; Goldstein A.; Bagg S.; Shekkarizfard M.; Hatzopoulou M. A Land Use Regression Model for Ambient Ultrafine Particles in Montreal, Canada: A Comparison of Linear Regression and a Machine Learning Approach. Environ. Res. 2016, 146, 65–72. 10.1016/j.envres.2015.12.016. 26720396

[ref15] Hatzopoulou M.; Valois M. F.; Levy I.; Mihele C.; Lu G.; Bagg S.; Minet L.; Brook J. Robustness of Land-Use Regression Models Developed from Mobile Air Pollutant Measurements. Environ. Sci. Technol. 2017, 51, 3938–3947. 10.1021/acs.est.7b00366. 28241115

[ref16] Apte J. S.; Messier K. P.; Gani S.; Brauer M.; Kirchstetter T. W.; Lunden M. M.; Marshall J. D.; Portier C. J.; Vermeulen R. C. H.; Hamburg S. P. High-Resolution Air Pollution Mapping with Google Street View Cars: Exploiting Big Data. Environ. Sci. Technol. 2017, 51, 6999–7008. 10.1021/acs.est.7b00891. 28578585

[ref17] Li H. Z.; Gu P.; Ye Q.; Zimmerman N.; Robinson E. S.; Subramanian R.; Apte J. S.; Robinson A. L.; Presto A. A. Spatially Dense Air Pollutant Sampling: Implications of Spatial Variability on the Representativeness of Stationary Air Pollutant Monitors. Atmos. Environ.: X 2019, 2, 100012 10.1016/j.aeaoa.2019.100012.

[ref18] Robinson E. S.; Shah R. U.; Messier K.; Gu P.; Li H. Z.; Apte J. S.; Robinson A. L.; Presto A. A. Land-Use Regression Modeling of Source-Resolved Fine Particulate Matter Components from Mobile Sampling. Environ. Sci. Technol. 2019, 53, 8925 10.1021/acs.est.9b01897. 31313910

[ref19] Hoek G. Methods for Assessing Long-Term Exposures to Outdoor Air Pollutants. Curr. Environ. Health Rep. 2017, 4, 450–462. 10.1007/s40572-017-0169-5. 29064065 PMC5676801

[ref20] Helmink A. S. v. d. Z. H. J. P. Meetresultaten Luchtkwaliteit Amsterdam 2018; GGD Amsterdam, 2019.

[ref21] de Smet P. A. M.; Visser S.; Valster N. L.; Schuch W. J. L.; Geijer M. N.; Wesseling J. P.; van den Beld W. A.; Drukker D.; Groot Wassink H.; Sanders A. Monitoringsrapportage NSL 2020; RIVM, 2020.

[ref22] Khan J.; Kakosimos K.; Raaschou-Nielsen O.; Brandt J.; Jensen S. S.; Ellermann T.; Ketzel M. Development and Performance Evaluation of New AirGIS – A GIS Based Air Pollution and Human Exposure Modelling System. Atmos. Environ. 2019, 198, 102–121. 10.1016/j.atmosenv.2018.10.036.

[ref23] Jensen S.; Ketzel M.; Khan J.; Valencia V.; Brandt J. .; Jesper H.; Lise M.; Plejdrup M. S.; Ellermann S. H. T. Videnskabelig Rapport Fra DCE-Nationalt Center for Miljø Og Energi LUFTEN PÅ DIN VEJ 2.0, 2021.

[ref24] Ellermann T.; Nordstrøm C.; Brandt J.; Christensen J.; Ketzel M.; Massling A.; Bossi R.; Marie L.; Camilla F.; Steen G.; Jensen S.; Nielsen O.-K.; Winther M.; Poulsen M. B.; Nygaard J.; Klenø Nøjgaard J. Videnskabelig Rapport Fra DCE-Nationalt Center for Miljø Og Energi, 2021.

[ref25] Weichenthal S.; Van Ryswyk K.; Goldstein A.; Shekarrizfard M.; Hatzopoulou M. Characterizing the Spatial Distribution of Ambient Ultrafine Particles in Toronto, Canada: A Land Use Regression Model. Environ. Pollut. 2016, 208, 241–248. 10.1016/j.envpol.2015.04.011. 25935348

[ref26] Shairsingh K. K.; Jeong C.-H.; Wang J. M.; Brook J. R.; Evans G. J. Urban Land Use Regression Models: Can Temporal Deconvolution of Traffic Pollution Measurements Extend the Urban LUR to Suburban Areas?. Atmos. Environ. 2019, 196, 143–151. 10.1016/j.atmosenv.2018.10.013.

[ref27] Jerrett M.; Arain M. A.; Kanaroglou P.; Beckerman B.; Crouse D.; Gilbert N. L.; Brook J. R.; Finkelstein N.; Finkelstein M. M. Modeling the Intraurban Variability of Ambient Traffic Pollution in Toronto, Canada. J. Toxicol. Environ. Health, Part A 2007, 70, 200–212. 10.1080/15287390600883018. 17365582

[ref28] Noth E. M.; Hammond S. K.; Biging G. S.; Tager I. B. A Spatial-Temporal Regression Model to Predict Daily Outdoor Residential PAH Concentrations in an Epidemiologic Study in Fresno, CA. Atmos. Environ. 2011, 45, 2394–2403. 10.1016/j.atmosenv.2011.02.014.

[ref29] Johnson M.; Macneill M.; Grgicak-Mannion A.; Nethery E.; Xu X.; Dales R.; Rasmussen P.; Wheeler A. Development of Temporally Refined Land-Use Regression Models Predicting Daily Household-Level Air Pollution in a Panel Study of Lung Function among Asthmatic Children. J. Expo. Sci. Environ. Epidemiol. 2013, 23, 259–267. 10.1038/jes.2013.1. 23532094

[ref30] Xu J.; Yang W.; Han B.; Wang M.; Wang Z.; Zhao Z.; Bai Z.; Vedal S. An Advanced Spatio-Temporal Model for Particulate Matter and Gaseous Pollutants in Beijing, China. Atmos. Environ. 2019, 211, 120–127. 10.1016/j.atmosenv.2019.04.011.

[ref31] Wang J.; Cohan D. S.; Xu H. Spatiotemporal Ozone Pollution LUR Models: Suitable Statistical Algorithms and Time Scales for a Megacity Scale. Atmos. Environ. 2020, 237, 117671 10.1016/j.atmosenv.2020.117671.

[ref32] Kerckhoffs J.; Hoek G.; Messier K. P.; Brunekreef B.; Meliefste K.; Klompmaker J. O.; Vermeulen R. Comparison of Ultrafine Particles and Black Carbon Concentration Predictions from a Mobile and Short-Term Stationary Land-Use Regression Model. Environ. Sci. Technol. 2016, 50, 12894–12902. 10.1021/acs.est.6b03476. 27809494

[ref33] Karner A. A.; Eisinger D. S.; Niemeier D. A. Near-Roadway Air Quality: Synthesizing the Findings from Real-World Data. Environ. Sci. Technol. 2010, 44, 5334–5344. 10.1021/es100008x. 20560612

[ref34] Beckerman B.; Jerrett M.; Brook J. R.; Verma D. K.; Arain M. A.; Finkelstein M. M. Correlation of Nitrogen Dioxide with Other Traffic Pollutants near a Major Expressway. Atmos. Environ. 2008, 42, 275–290. 10.1016/j.atmosenv.2007.09.042.

[ref35] McAdam K.; Steer P.; Perrotta K. Using Continuous Sampling to Examine the Distribution of Traffic Related Air Pollution in Proximity to a Major Road. Atmos. Environ. 2011, 45, 2080–2086. 10.1016/j.atmosenv.2011.01.050.

[ref36] Richmond-Bryant J.; Chris Owen R.; Graham S.; Snyder M.; McDow S.; Oakes M.; Kimbrough S. Estimation of On-Road NO2 Concentrations, NO2/NOX Ratios, and Related Roadway Gradients from near-Road Monitoring Data. Air Qual., Atmos. Health 2017, 10, 611–625. 10.1007/s11869-016-0455-7. 30245748 PMC6145484

[ref37] Kerckhoffs J.; Hoek G.; Portengen L.; Brunekreef B.; Vermeulen R. C. H. Performance of Prediction Algorithms for Modeling Outdoor Air Pollution Spatial Surfaces. Environ. Sci. Technol. 2019, 53, 1413–1421. 10.1021/acs.est.8b06038. 30609353

