# Supplementary material for: Mixed-Effects Modeling Framework for Amsterdam and Copenhagen for Outdoor NO2 Concentrations Using Measurements Sampled with Google Street View Cars
Source: Environ Sci Technol. 2022 Mar 9;56(11):7174–84. doi: 10.1021/acs.est.1c05806 (PMC9178915; doi:10.1021/acs.est.1c05806)
Supplement: Supplementary file 1 — es1c05806_si_001.pdf [file es1c05806_si_001.pdf]

Supporting Information for:

A Mixed-effects Modelling Framework for Amsterdam and Copenhagen for Outdoor NO_2 Concentrations using Measurements Sampled with Google Street View Cars

Jules Kerckhoffs[1,], Jibran Khan[2,3], Gerard Hoek[1], Zhendong Yuan[1], Thomas Ellermann[2], Ole Hertel[4], Matthias Ketzel[2,5], Steen Solvang Jensen[2], Kees Meliefste[1], Roel Vermeulen[1,6]*

[1]Institute for Risk Assessment Sciences, Utrecht University, 3584 CK Utrecht, Netherlands
[2]Department of Environmental Science, Aarhus University, DK-4000 Roskilde, Denmark
[3]Danish Big Data Centre for Environment and Health (BERTHA), Aarhus University, DK-4000 Roskilde, Denmark
[4]Department of Bioscience, Aarhus University, DK-4000 Roskilde, Denmark
[5]Global Centre for Clean Air Research (GCARE), University of Surrey, Guildford GU2 7XH, United Kingdom
[6]Julius Centre for Health Sciences and Primary Care, University Medical Centre, University of Utrecht, 3584 CK Utrecht, The Netherlands

*Corresponding author

General:
S1. Temporal correction
S2. LUR model Development

Amsterdam:
Table A1: GIS predictors for AMS LUR model
Table A2: LUR Model for AMS
Figure A1: High-resolution version of Figure 2 in main text.
Figure A2: Distribution of measurements and predictions with equal numbers of observations
Figure A3: Comparison between Data-Only, Mixed model estimates and LML
Figure A4: Scatterplots and Bland-Altman plots for all AAV comparisons with external datasets

Copenhagen:
Table B1: GIS predictors for CPH LUR model
Table B2: LUR Model for CPH
Figure B1: High-resolution version of Figure 3 in main text.
Figure B2: Distribution of measurements and predictions with equal numbers of observations
Figure B3: Comparison between Data-Only, Mixed model estimates and NOVANA
Figure B4: Scatterplots and Bland-Altman plots for all CAV comparisons with external datasets

S1. Temporal Correction

As street segments were measured at different times of the day and week, we applied a temporal correction to the measured data using nearby reference stations (one per city). Two correction methods were employed, the difference correction and the ratio correction. For AMS, we used the difference correction method to correct the spatial data, as done previously in mobile monitoring studies, e.g. Kerckhoffs et al. [1,2]. The reference station we used for this study is part of the Dutch National Air Quality Monitoring Program, and located in a suburban area of Amsterdam, away from traffic sources [3]. For CPH, we corrected the measured data using references values from a background air pollution monitoring station, located at the rooftop of the H.C. Ørsted Institute, University of Copenhagen, and part of the Danish Air Quality Monitoring Program [4]. This reference station is located on a rooftop, measuring very stable NO_2 concentrations, not influenced by traffic peaks. Therefore, for CPH, we applied a ratio correction method.

Both methods start with calculating the overall mean concentration of the entire campaign at the reference site. Then, a moving average of 4-hours at the reference site was calculated. In AMS, for each second, we subtracted the 4-hour moving average from the overall mean concentration. In CPH, we divided the 4-hour moving average by the overall mean concentration. Finally, this calculated value was added to the measured data for AMS and was used as a multiplier for CPH. Previous studies have used similar approaches concerning uncorrected, difference and ratio-corrected concentration levels [5]. We winsorised the measured data to the 2.5th and the 97.5th percentile. Here, values below the 2.5th percentile and above the 97.5th percentile were “replaced” by the respective percentile values of the original distribution. This reduces the influence of possibly spurious “extreme” outliers [6].

S2. LUR model development

For each 50-m street segment, we acquired GIS predictors for LUR modelling. An overview of GIS predictors is provided in Supplementary Tables A1 and B1. In summary, a range of traffic variables was defined, including traffic intensity (number of vehicles/day) and road length variables in 50 – 1000 m buffers. Moreover, the data on land use, e.g., port, industry, urban green, airports, and population density in buffers of 100 to 5000 m, was included as the LUR model inputs. For CPH, the data on estimated building heights (m) was included as input (25-, 50- and 100-meters buffers) to the LUR model.

Traffic counts were acquired from the national traffic databases in the Netherlands [7] and Denmark [8]. Land use variables were obtained from the European CORINE database [9], and population density variables were retrieved from the Netherlands Environmental Assessment Agency [10]. For CPH, building footprints data was provided by the Department of Environmental Science, Aarhus University, Roskilde, Denmark, originally acquired from the Danish Geodata Agency. The building height is estimated for each building, using the national elevation model having a resolution of 1 m x 1 m (see Khan et al. [11] for more details).

The variable selection was performed using a supervised forward stepwise regression approach which starts with an empty (intercept only) model and then adds variables based on goodness of fit determined by the adjusted R^2 value [1,2]. The direction of the effect for all variables was determined before model development (see Tables A1 and B1), and the variable with the highest adjusted R^2 was included first in the model. The process of model building was stopped when new variables were not able to improve the adjusted R^2. The variables in the resulting models were checked for p-value and collinearity. That is, variables with a p-value > 0.10 and variance inflation factor > 3 were removed. LUR models were then used as “fixed” effects in the mixed model, described in the following subsection.

Table A1. Spatial predictor variables with units, a priori defined directions of effect, and buffer sizes in AMS.

Predictor variable	Abbreviation	Units	Direction of effect	Buffer	10th percentile	Mean	90th Percentile
**Agricultural land area[1]**	AGRI_	m^2	-	**100**	0	1159	0
				**300**	0	11288	0
				**500**	0	34779	64052
				**1000**	0	178942	649727
				**5000**	1710991	13290916	30287613
**Airport area[1]**	AIR_	m^2	+	**5000**	0	640794	1628347
**Industry area[1]**	INDUS_	m^2	+	**100**	0	2006	0
				**300**	0	17769	41347
				**500**	0	49074	180131
				**1000**	0	199831	730248
				**5000**	2902185	5182292	8091464
**Natural and forested areas[1]**	NATUR_	m^2	-	**100**	0	0	0
				**300**	0	0	0
				**500**	0	0	0
				**1000**	0	0	0
				**5000**	0	429332	2388024
**Port area[1]**	PORT_	m^2	+	**100**	0	2648	2516
				**300**	0	22447	85979
				**500**	0	59770	258949
				**1000**	0	226393	956817
				**5000**	0	6167718	13267782
**Residential land area[1]**	RES_	m^2	+	**100**	0	21152	31416
				**300**	0	183950	282742
				**500**	0	492795	785396
				**1000**	104805	1826998	3040804
				**5000**	17043122	32157817	46811645
**Transportation area[1]**	TRANS_	m^2	+	**100**	0	0	0
				**300**	0	0	0
				**500**	0	13457	2874
				**1000**	0	50061	233924
				**5000**	519010	1340506	2093995
**Urban Green area[1]**	URBG_	m^2	-	**100**	0	2351	7952
				**300**	0	25250	102385
				**500**	0	79234	268822
				**1000**	0	361378	852803

				**5000**	6248972	9362179	13883066
**Water**[1]	WATER_	m^2		**100**	0	0	0
				**300**	0	10238	30716
				**500**	0	37897	152537
				**1000**	0	222478	741893
				**5000**	2904140	8362718	12310634
**Population density**[2]	POP_	n	+	**100**	0	319	770
				**300**	0	2385	5355
				**500**	25	6091	13410
				**1000**	1485	21762	46115
				**5000**	146514	366674	580006
**Traffic intensity on nearest road**[3]	TRAFNEAR	Veh/day	+		137	9986	22487
**Traffic intensity on nearest major road**[3]	TRAFMAJOR	Veh/day	+		3115	14212	28042
**Heavy-duty traffic intensity on nearest road**[3]	HTRAFNEAR	Veh/day	+		0	619	1333
**Heavy-duty traffic intensity on nearest major road**[3]	HTRAFMAJOR	Veh/day	+		54	980	2138
**Road length of all roads**[3]	RDL_	m	+	**25**	39	66	100
				**50**	100	187	294
				**100**	254	636	966
				**300**	2029	4880	7083
				**500**	7893	13761	19090
				**1000**	29092	50344	68095
**Road length of all major roads**[3]	MRDL_	m	+	**25**	0	24	91
				**50**	0	69	198
				**100**	0	210	534
				**300**	0	1532	3554
				**500**	0	3787	8800
				**1000**	3015	13627	31882
**Traffic intensity on all roads**	TLOA_	(Veh/day)*m	+	**25**	0	634722	1579945
**(sum of (traffic intensity * length of all segments))** [3]				**50**	6393	1815133	4797110
				**100**	78159	5494027	14290643
				**300**	3038453	36918277	1.01E+08
				**500**	13741938	44180744	87625101
				**1000**	62971674	166892417	324383228
**Traffic intensity on all major roads**	TMLOA_	(Veh/day)*m	+	**25**	0	543194	1387293
**(sum of (traffic intensity* length of all segments))** [3]				**50**	0	1548534	4473085
				**100**	0	4550786	13415227
				**300**	0	12783795	26453420

				**500**	0	30877644	64537936
				**1000**	26948221	116664854	238567679
**Heavy-duty traffic intensity on all roads** **(sum of (heavy-duty traffic intensity* length of all segments))** [3]	HLOA_	(Veh/day)*m	+	**25**	0	40889	59383
				**50**	0	115926	205867
				**100**	1607	336918	909792
				**300**	84212	2085219	8507687
				**500**	387896	2664654	6027990
				**1000**	2550331	10515869	25699398
**Heavy-duty traffic intensity on major roads** **(sum of (heavy-duty traffic intensity*length of all segments)** [3]	HMLOA_	(Veh/day)*m	+	**25**	0	35363	41295
				**50**	0	100488	141074
				**100**	0	286355	742416
				**300**	0	846066	1614863
				**500**	0	2033997	4769469
				**1000**	966747	8018542	22978460
				**100**	0	35	0
**Highway**[4]	HIGH_	m	+	**500**	0	469	1977
				**10000**	0	1665	4185
**Railway**[4]	RAIL_	m	+	**100**	0	50	0
				**500**	0	1380	4734
				**10000**	0	5695	20657
				**100**	0	1	2
**Restaurants**[4]	REST_	n	+	**500**	0	16	39
				**1000**	1	61	164
				**100**	0	0	0
**Traffic Lights**[4]	TL_	n	+	**500**	0	6	18
				**1000**	0	24	57
				**100**	0	0	1
**Bus Stops**[4]	BS_	n	+	**500**	0	6	11
				**1000**	7	21	35

[1]Source: CORINE (Copernicus Land Monitoring Service) 2018, [2]Source: CBS (Central Bureau of Statistics Netherlands) 2017, [3]Source: NWB (National Road Network Netherlands) 2017, [4]Source: OSM (Open Street Map) 2017.

Table A2: An overview of GIS predictors for the LUR model based on GSV measurements in AMS.

Variable	Estimate	StDev	Pvalue
Intercept	6.96	0.09	<0.001
Traffic intensity on major roads in a 50-m buffer	1.58*	0.05	<0.001
Length of major roads in a 100-m buffer	1.39	0.11	<0.001
Population density in a 5000-m buffer	2.13	0.06	<0.001
Traffic intensity on the nearest road	1.31	0.04	<0.001
Area of ports in a 1000-m buffer	0.58	0.04	<0.001
Traffic intensity on all roads in a 500-m buffer	2.00	0.07	<0.001
Length of major roads in a 50-m buffer	2.20	0.10	<0.001
Area of transportation services in a 5000-m buffer	1.29	0.06	<0.001
Area of ports in a 5000-m buffer	1.03	0.07	<0.001
R^2 = 0.485			

**Regression slopes and standard error (between brackets) are multiplied by the difference between the 10th and 90th percentile for all variables.*

Figure A1. High resolution version of figure 2a-e in main text: Maps of measurements, predictions, and variance in Amsterdam. a) Data-only map, b) Standard error of the mean, c) LUR model (fixed effects), d) Mixed-effect model and e) random components.

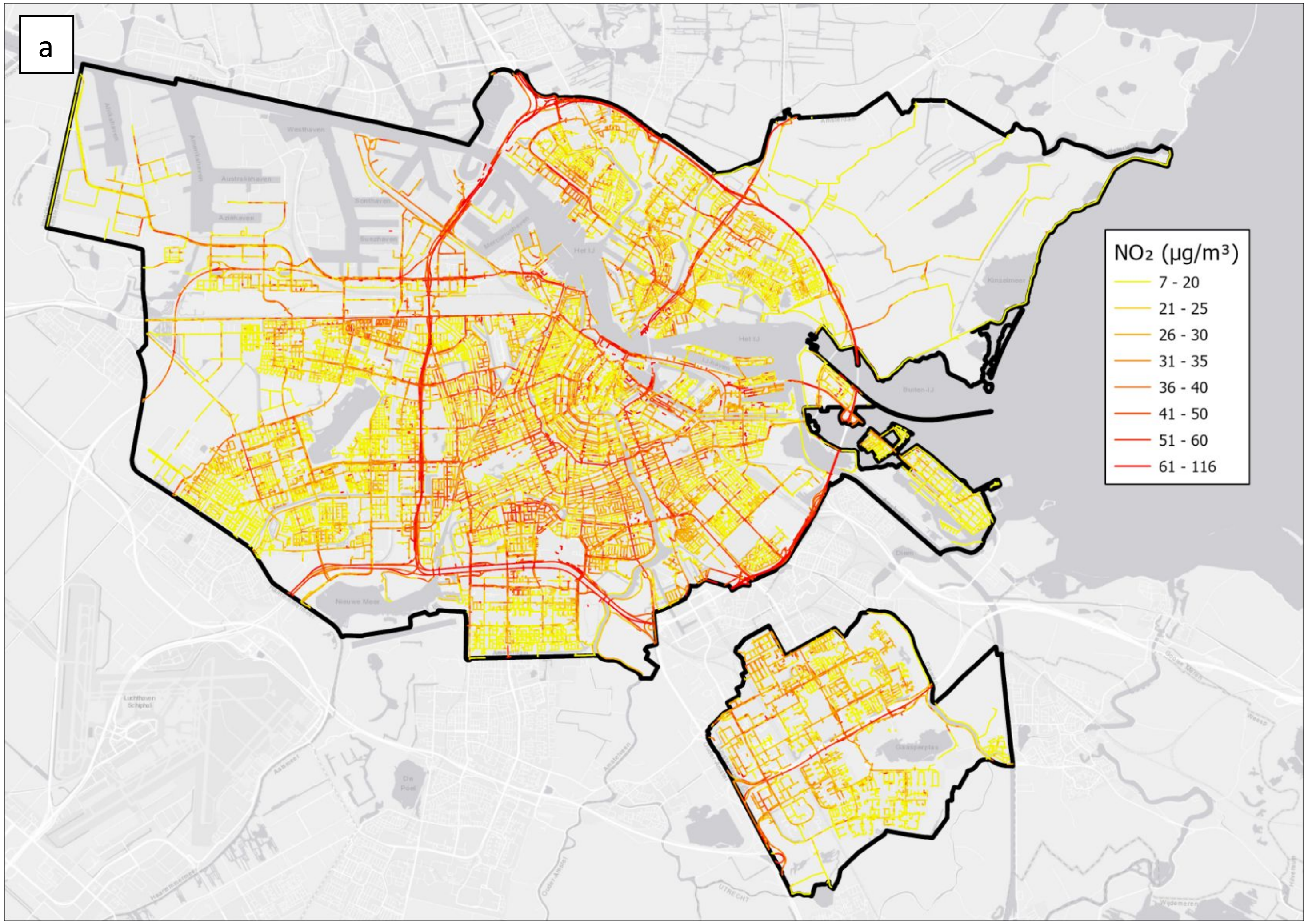

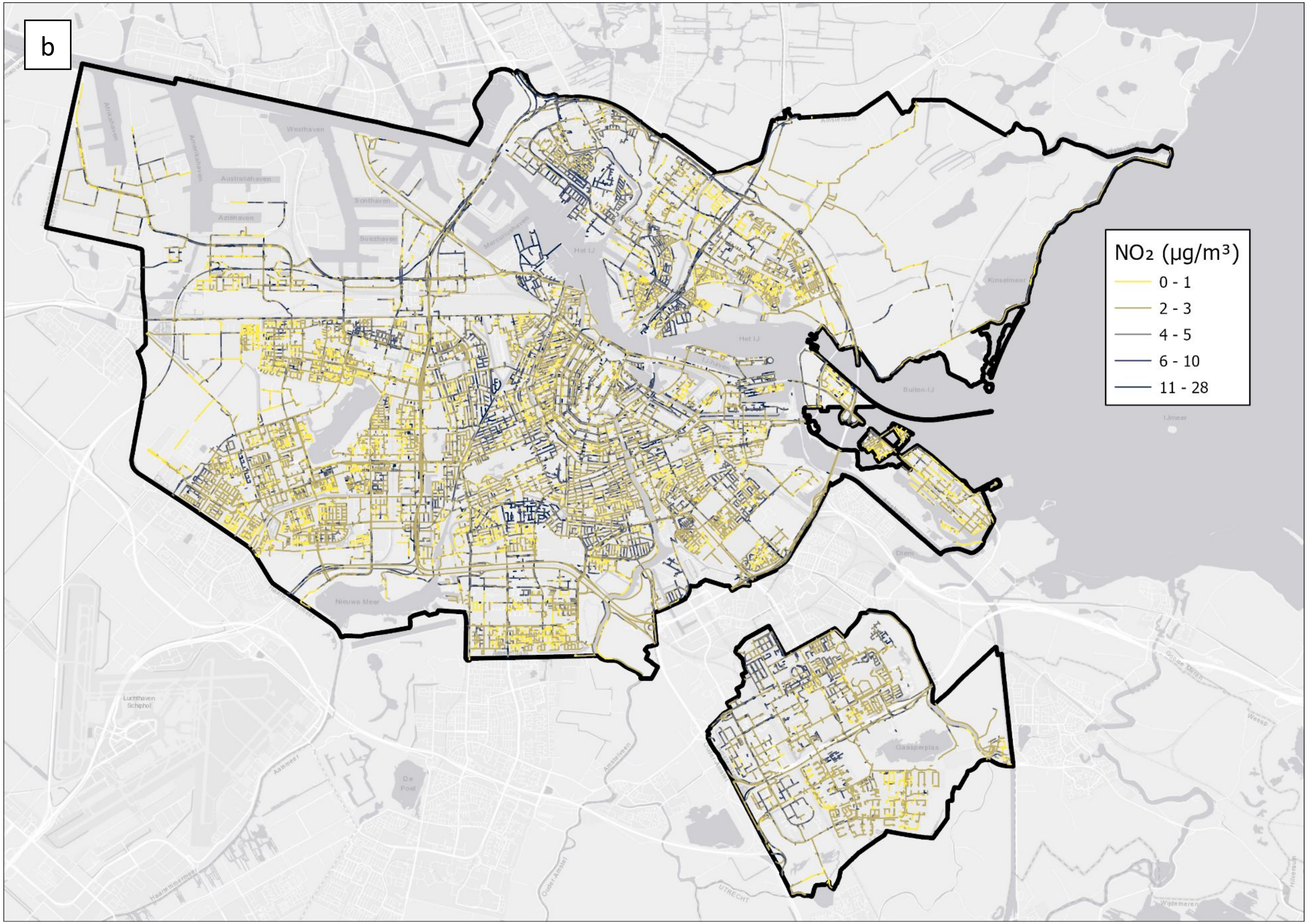
b
NO₂ (μg/m³)
0 - 1
2 - 3
4 - 5
6 - 10
11 - 28

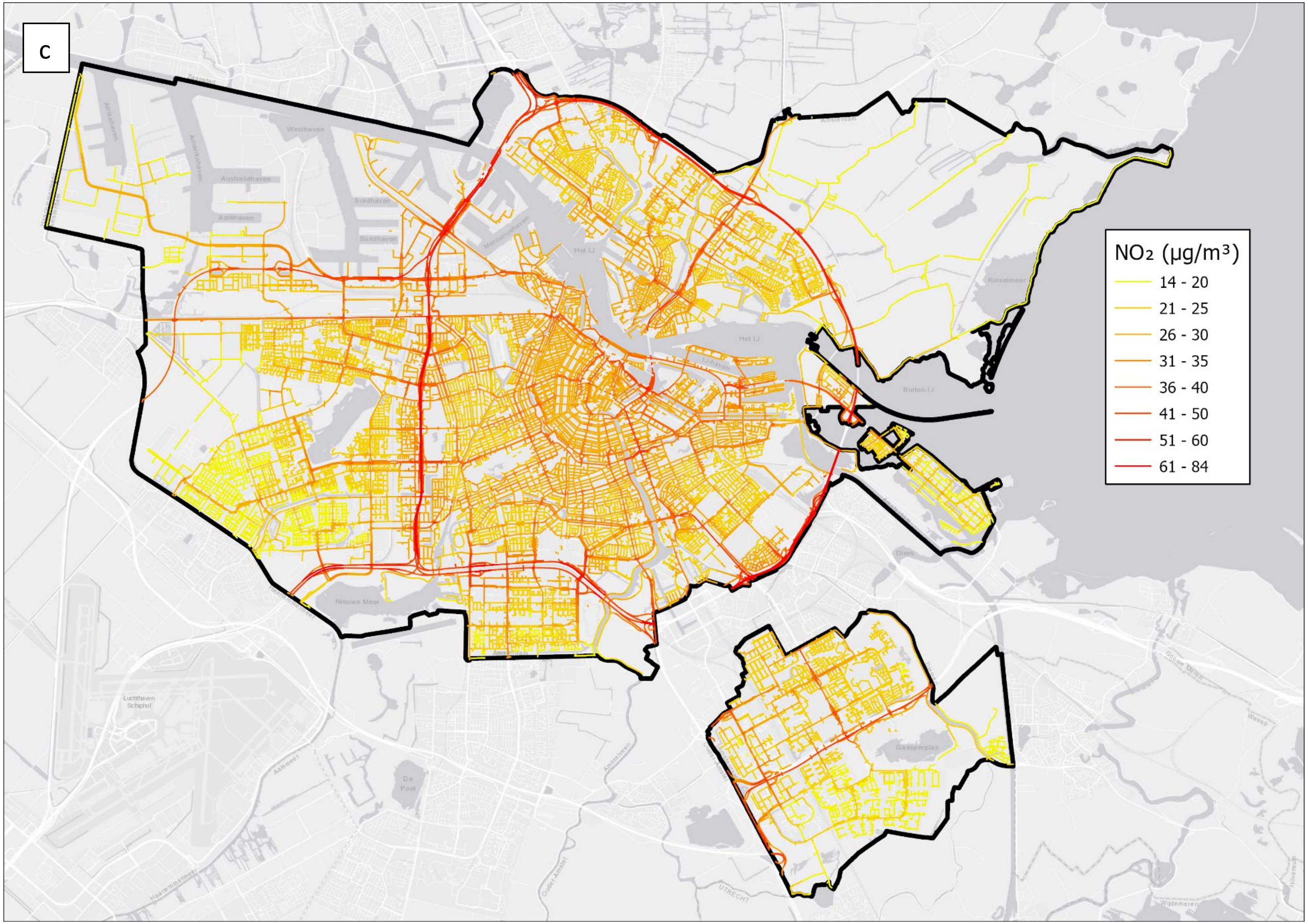
c
NO_2 ($\mu g/m^3$)
14 - 20
21 - 25
26 - 30
31 - 35
36 - 40
41 - 50
51 - 60
61 - 84
Westhaven
Het IJ
Kinselmeer
Buiten-IJ
Nieuwe Meer
Gaasperplas

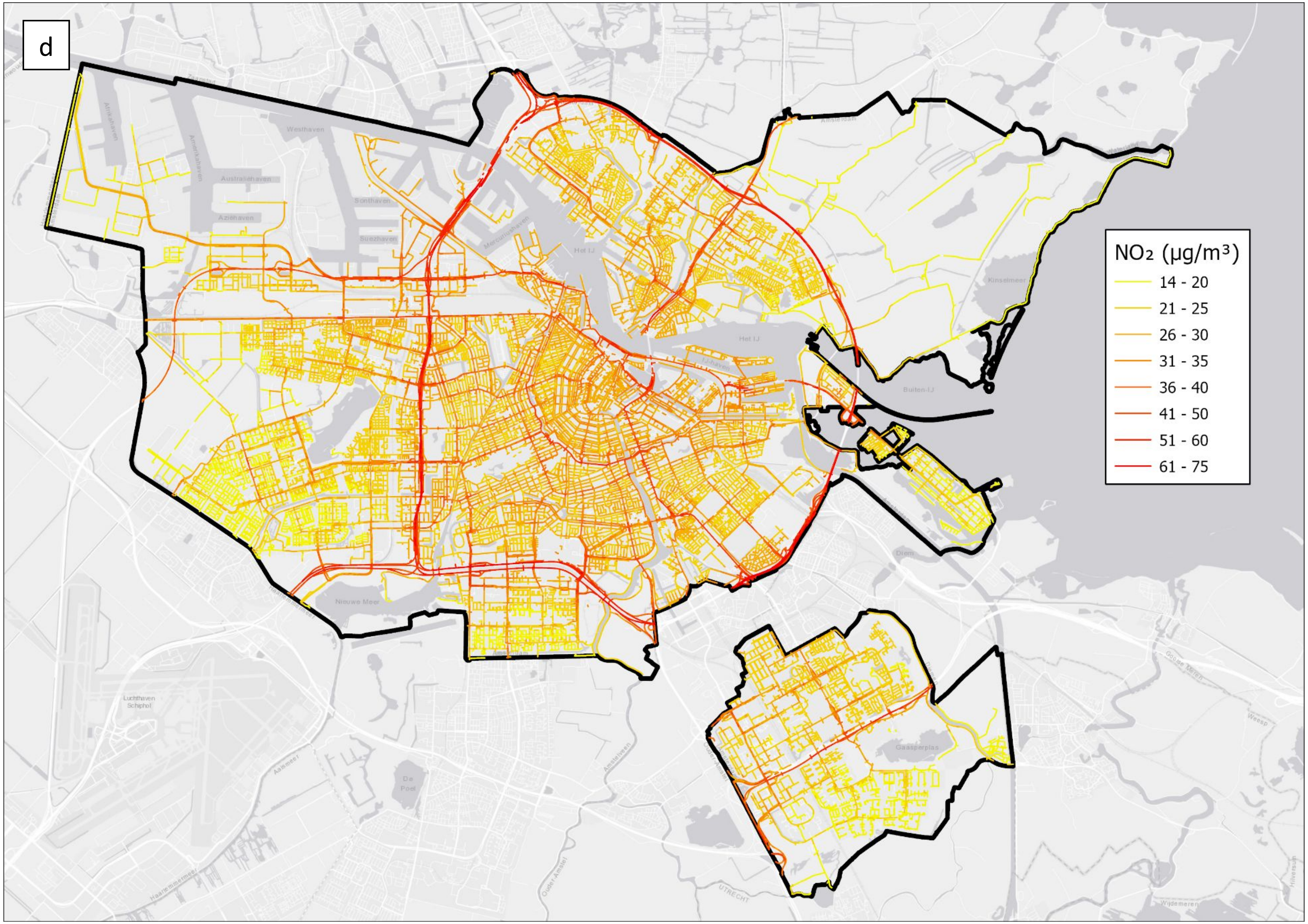
d
NO_2 (µg/m³)
14 - 20
21 - 25
26 - 30
31 - 35
36 - 40
41 - 50
51 - 60
61 - 75

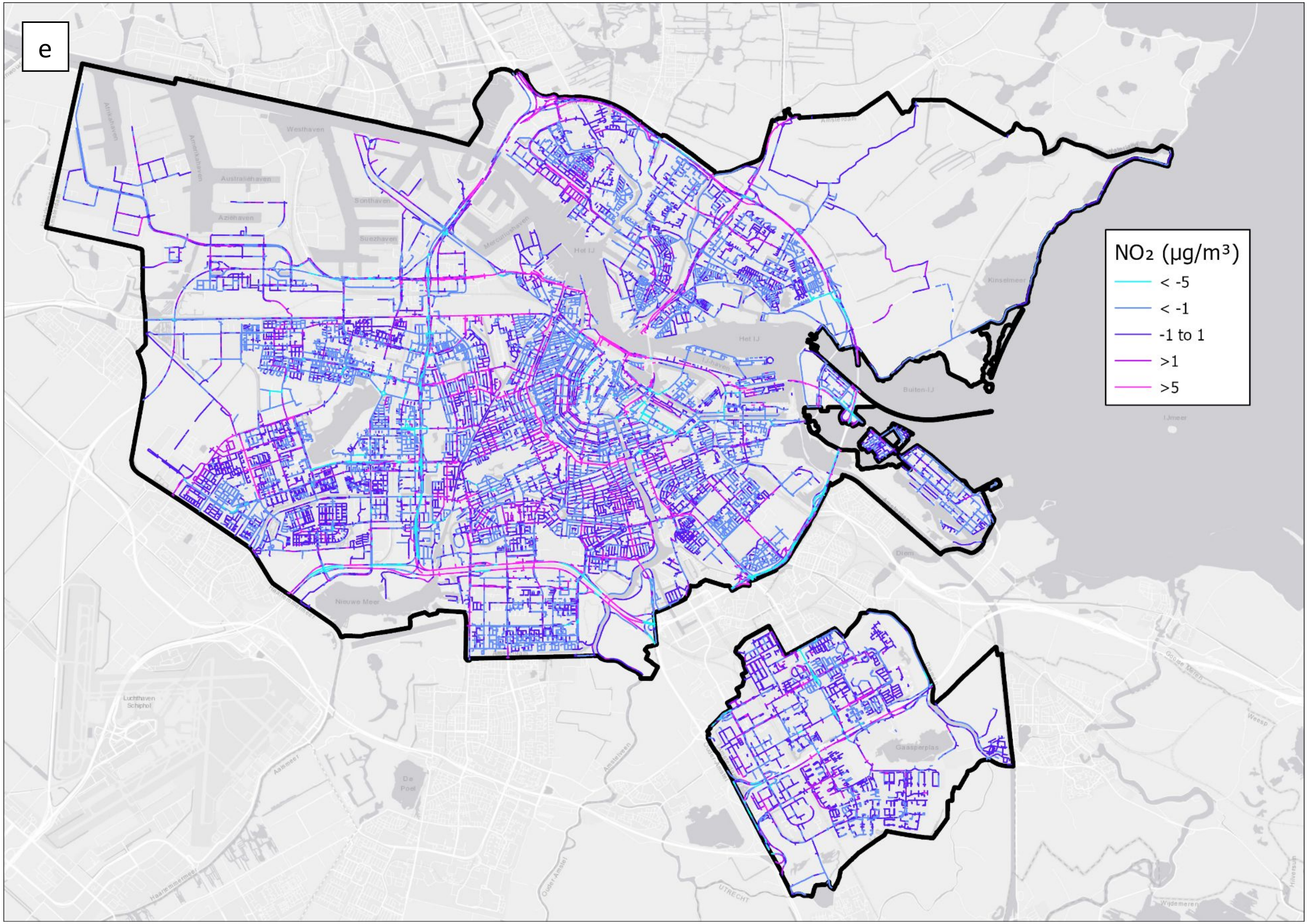
e
NO2 (μg/m3)
< -5
< -1
-1 to 1
>1
>5

Figure A2: Distribution of measurements and predictions from the AAV and measurements for the external datasets (top). Subsequent boxplots represent the distributions with an equal number of sites between the AAV and external comparison dataset.

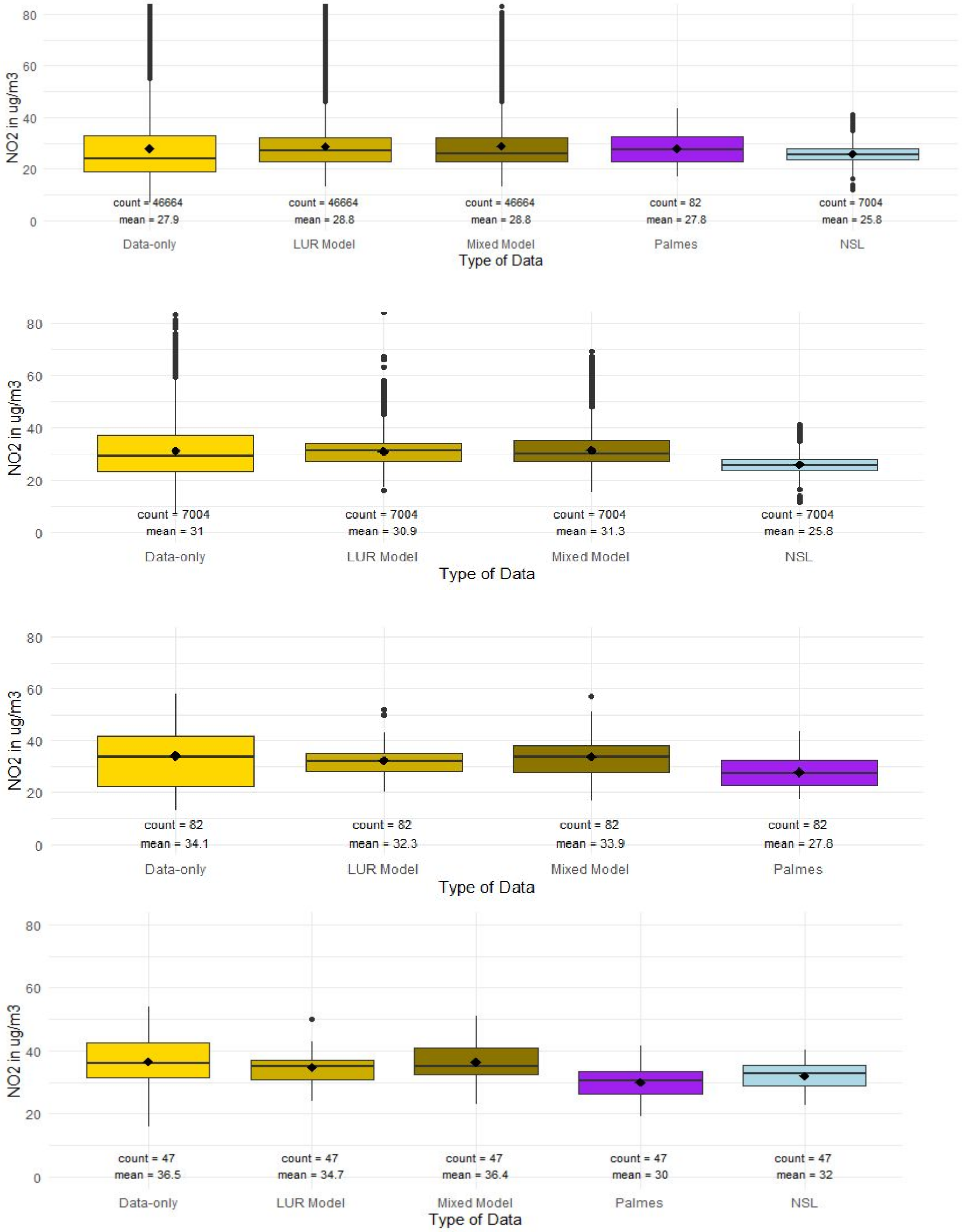

Figure A3: Scatterplot of AAV concentrations nearby routine measurement stations.

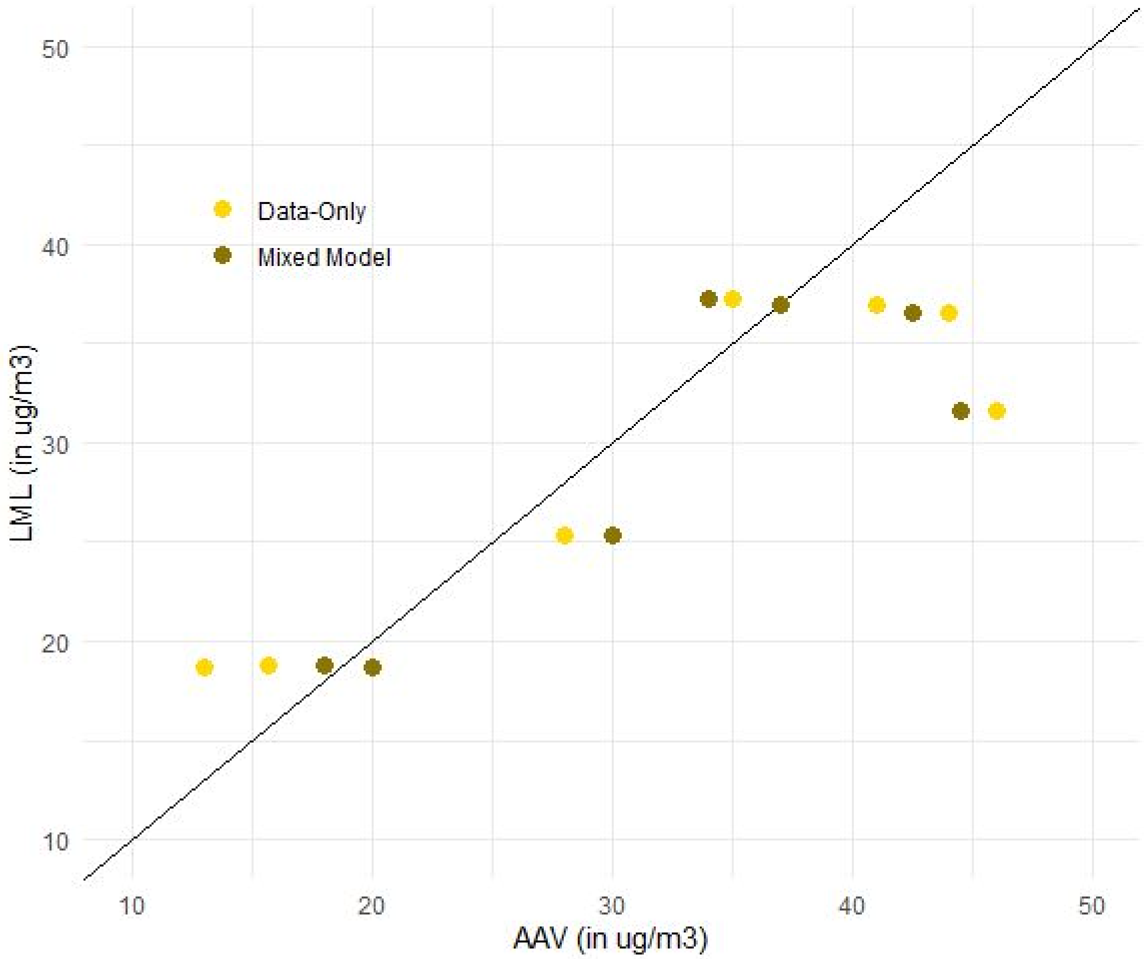

Figure A4: Scatterplots and Bland-Altman plots for comparisons between GSV method and a) Palmes measurements (n=82), b) NSL national model predictions (n=7,004) and c) Palmes measurements with both GSV and NSL predictions available (n=47).

a)

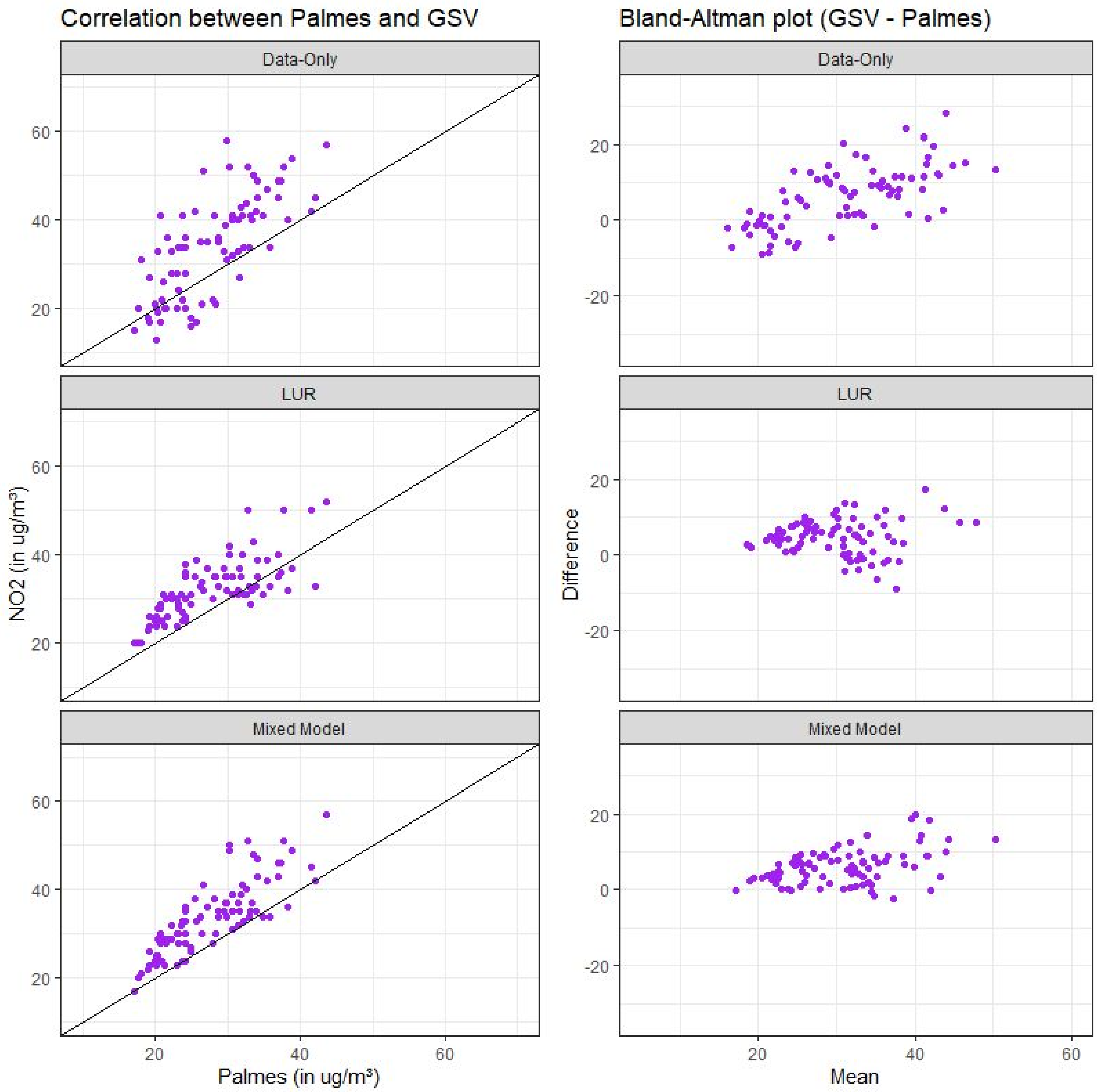

b)

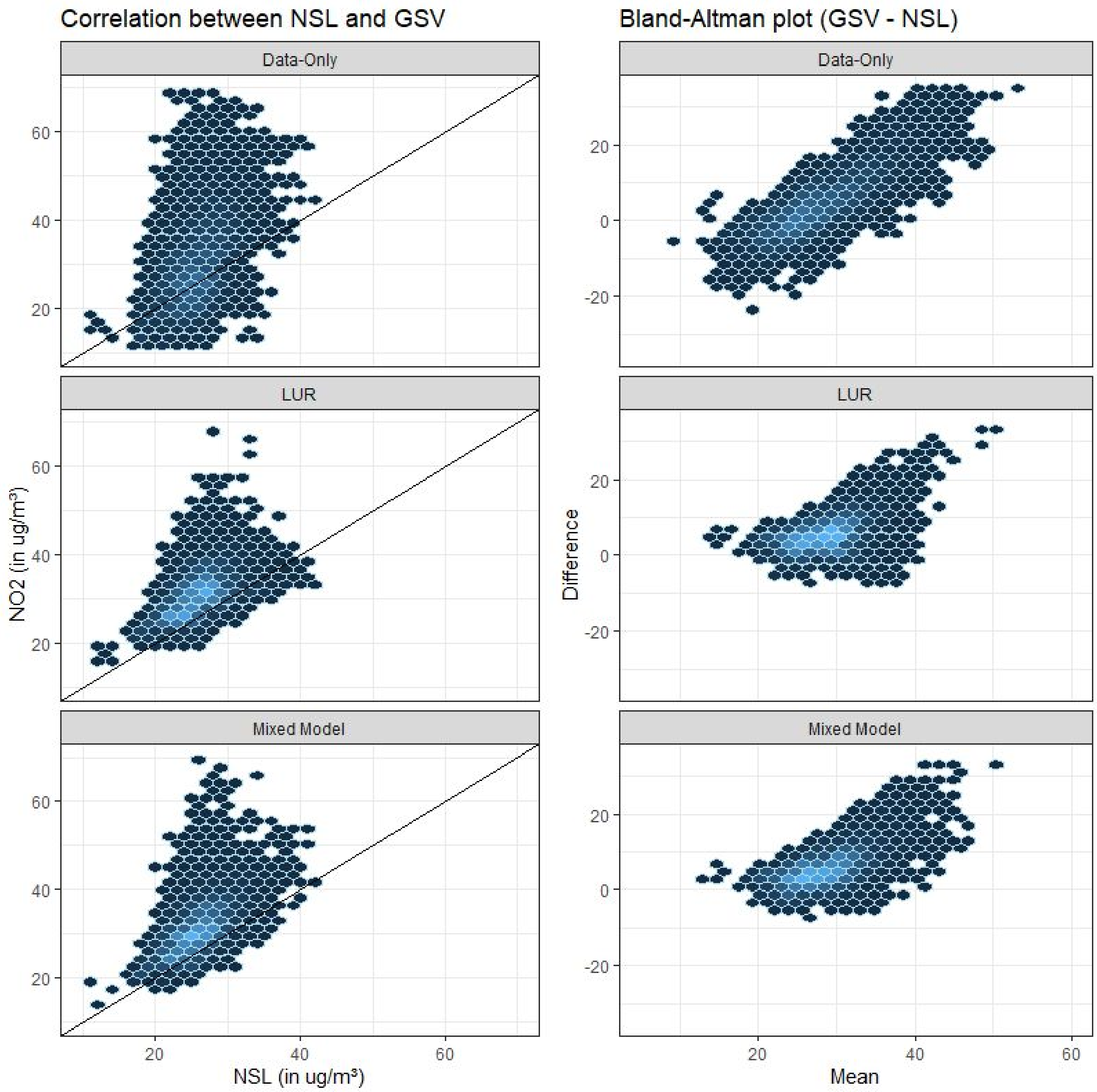

**Values are divided into hexagons with the light blue colour representing more points.*

123 **c)**

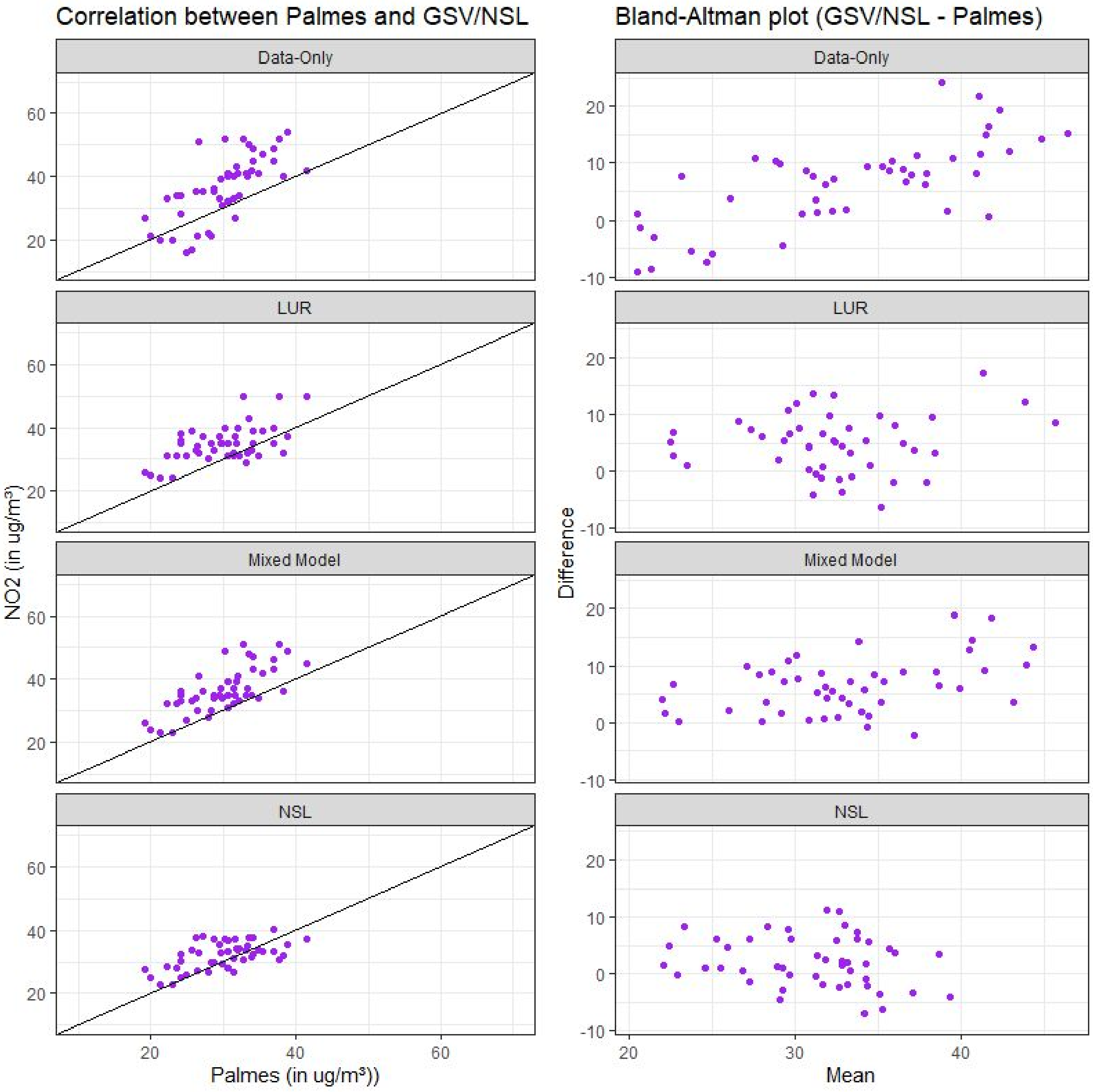
Correlation between Palmes and GSV/NSL
Bland-Altman plot (GSV/NSL - Palmes)
Data-Only
LUR
Mixed Model
NSL
NO2 (in ug/m³)
Palmes (in ug/m³))
Difference
Mean

124

Table B1. Spatial predictor variables with units, a priori defined directions of effect, and buffer sizes in CPH.

Predictor variable	Abbreviation	Units	Direction of effect	Buffer	10th percentile	Mean	90th Percentile
**Agricultural land area**[1]	AGRI_	m^2	-	**100**	0	388	0
				**300**	0	3755	0
				**500**	0	11235	0
				**1000**	0	50079	0
				**5000**	0	1639222	6052512
**Airport area**[1]	AIR_	m^2	+	**5000**	0	2279059	10257996
**Industry area**[1]	INDUS_	m^2	+	**100**	0	1734	0
				**300**	0	15683	47134
				**500**	0	43180	170951
				**1000**	0	169084	581165
				**5000**	2226557	4831986	8521951
**Port area**[1]	PORT_	m^2	+	**100**	0	1302	0
				**300**	0	12876	5189
				**500**	0	38070	109433
				**1000**	0	168154	621898
				**5000**	0	5497139	11394038
**Residential land area**[1]	RES_	m^2	+	**100**	0	24795	31416
				**300**	16158	213730	282742
				**500**	132316	573058	785396
				**1000**	882895	2155900	3085851
				**5000**	24183197	40917979	56817635
**Transportation area**[1]	TRANS_	m^2	+	**100**	0	0	0
				**300**	0	0	0
				**500**	0	10360	0
				**1000**	0	41926	50605
				**5000**	20836	1162168	1968832
**Urban Green area**[1]	URBG_	m^2	-	**100**	0	2025	5996
				**300**	0	24073	91499
				**500**	0	78733	250768
				**1000**	0	376608	876317
				**5000**	6378671	9180538	11720888
**Water**[1]	WATER_	m^2		**100**	0	0	0
				**300**	0	2762	0
				**500**	0	10721	6488
				**1000**	0	68456	258501

				**5000**	1310084	9035349	22908911
**Population density**[2]	POP_	n	+	**100**	33	228	500
				**300**	124	678	1471
				**500**	223	1115	2377
				**1000**	480	2135	4165
				**5000**	2905	7325	10985
**Building Heights**[3]	BHEI_	m	+	**25**	0	10	19
				**50**	5	9	16
				**100**	5	9	15
**Traffic intensity on nearest road**[3]	TRAFNEAR	Veh/day	+		204	4280	14375
**Traffic intensity on nearest major road**[3]	TRAFMAJOR	Veh/day	+		4313	14031	29630
**Road length of all roads**[3]	RDL_	m	+	**25**	49	65	97
				**50**	100	172	263
				**100**	315	572	825
				**300**	2619	4439	5920
				**500**	6888	11662	15137
				**1000**	26167	42971	54871
**Road length of all major roads**[3]	MRDL_	m	+	**25**	0	15	50
				**50**	0	41	155
				**100**	0	118	391
				**300**	0	823	1858
				**500**	0	2132	4079
				**1000**	1864	7842	14680
**Traffic intensity on all roads (sum of (traffic intensity * length of all segments))** [3]	TLOA_	(Veh/day)*m	+	**25**	10030	324650	967861
				**50**	20391	853293	2385581
				**100**	92546	2478745	6768816
				**300**	1867503	16909530	38058226
				**500**	9900059	43678237	84379523
				**1000**	52385172	1.61E+08	2.81E+08
**Traffic intensity on all major roads (sum of (traffic intensity* length of all segments))** [3]	TMLOA_	(Veh/day)*m	+	**25**	0	253055	873472
				**50**	0	664742	2127245
				**100**	0	1877510	5731125
				**300**	0	12431834	31354988
				**500**	0	31921581	65642690
				**1000**	15251094	1.17E+08	2.21E+08

[1]*Source: CORINE (Copernicus Land Monitoring Service) 2018,* [2]*Source: EEA (European Environmental Agency)* [3]*Source:* Department of Environmental Science, Aarhus University*).*

Table B2: An overview of GIS predictors for the LUR model based on GSV measurements in CPH.

Variable	Estimate	StDev	Pvalue
Intercept	4.31	0.07	<0.001
Traffic intensity on all roads in a 50-m buffer	3.13	0.09	<0.001
Length of major roads in a 500-m buffer	1.06	0.10	<0.001
Traffic intensity on the nearest road	2.41	0.08	<0.001
Average building height in a 100-m buffer	1.31	0.06	<0.001
Area of transportation services in a 1000-m buffer	0.22	0.01	<0.001
Area of water in a 1000-m buffer	0.74	0.04	<0.001
Length of major roads in a 100-m buffer	1.36	0.09	<0.001
Area of transportation services in a 5000-m buffer	0.81	0.07	<0.001
Traffic intensity on all roads in a 300-m buffer	1.03	0.10	<0.001
R^2 = 0.541			

**Regression slopes and standard error (between brackets) are multiplied by the difference between 10th and 90th percentile for all variables.*

Figure B1: High resolution version of figure 3a-e in main text: Maps of measurements, predictions, and variance in Amsterdam. a) Data-only map, b) Standard error of the mean, c) LUR model (fixed effects), d) Mixed-effect model and e) random components

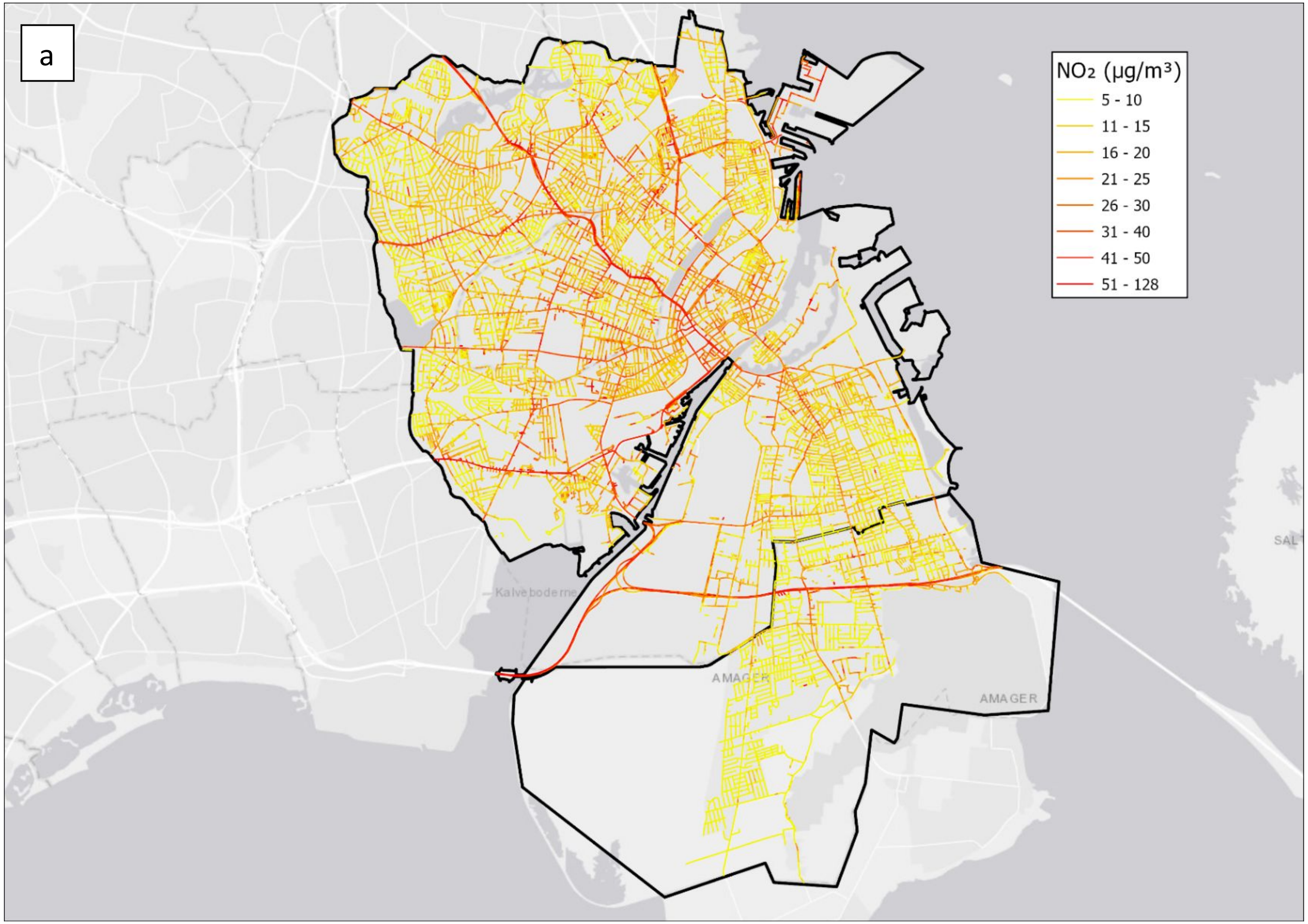

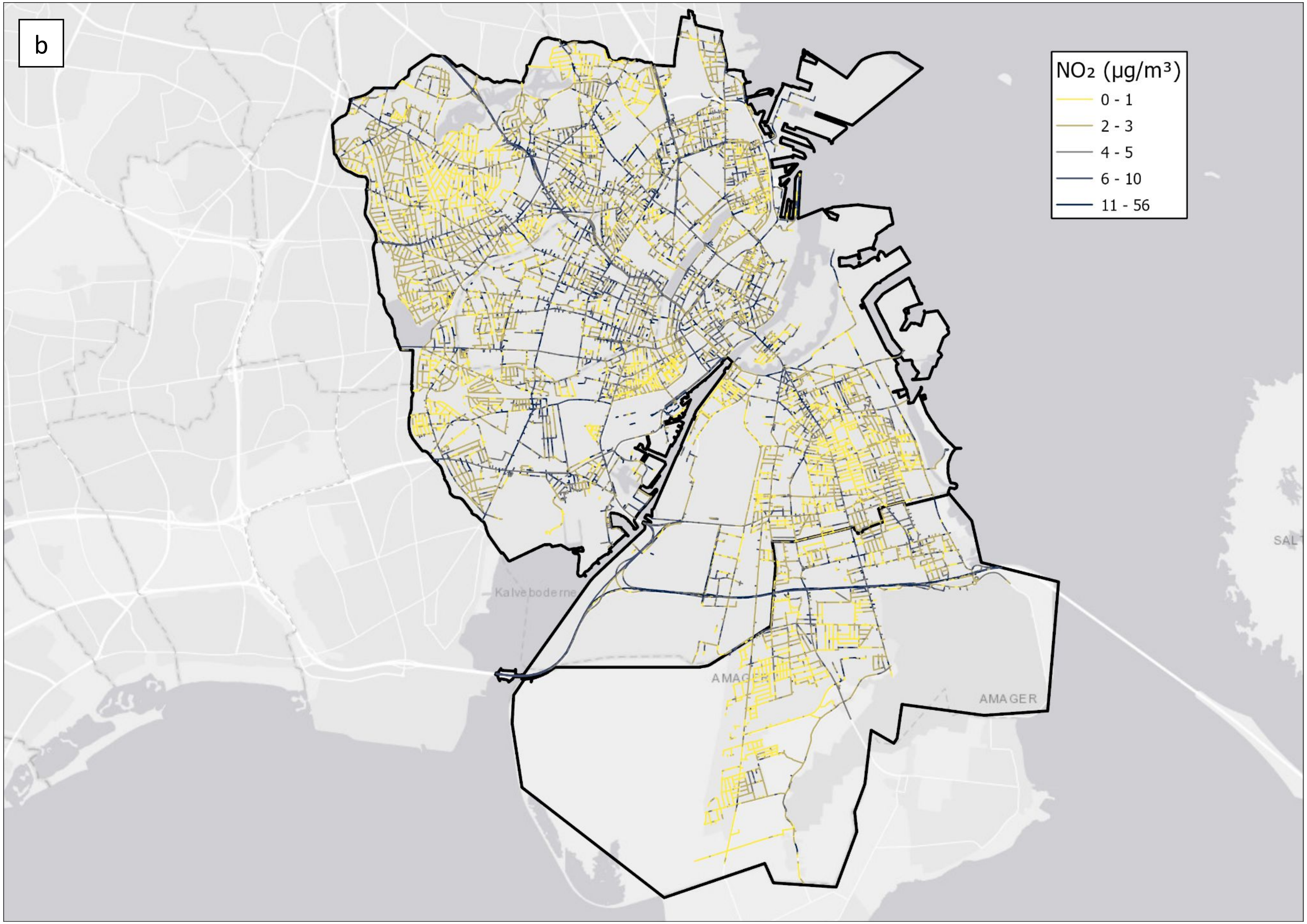
b
NO₂ (µg/m³)
0 - 1
2 - 3
4 - 5
6 - 10
11 - 56
Kalveboderne
AMAGER
AMAGER

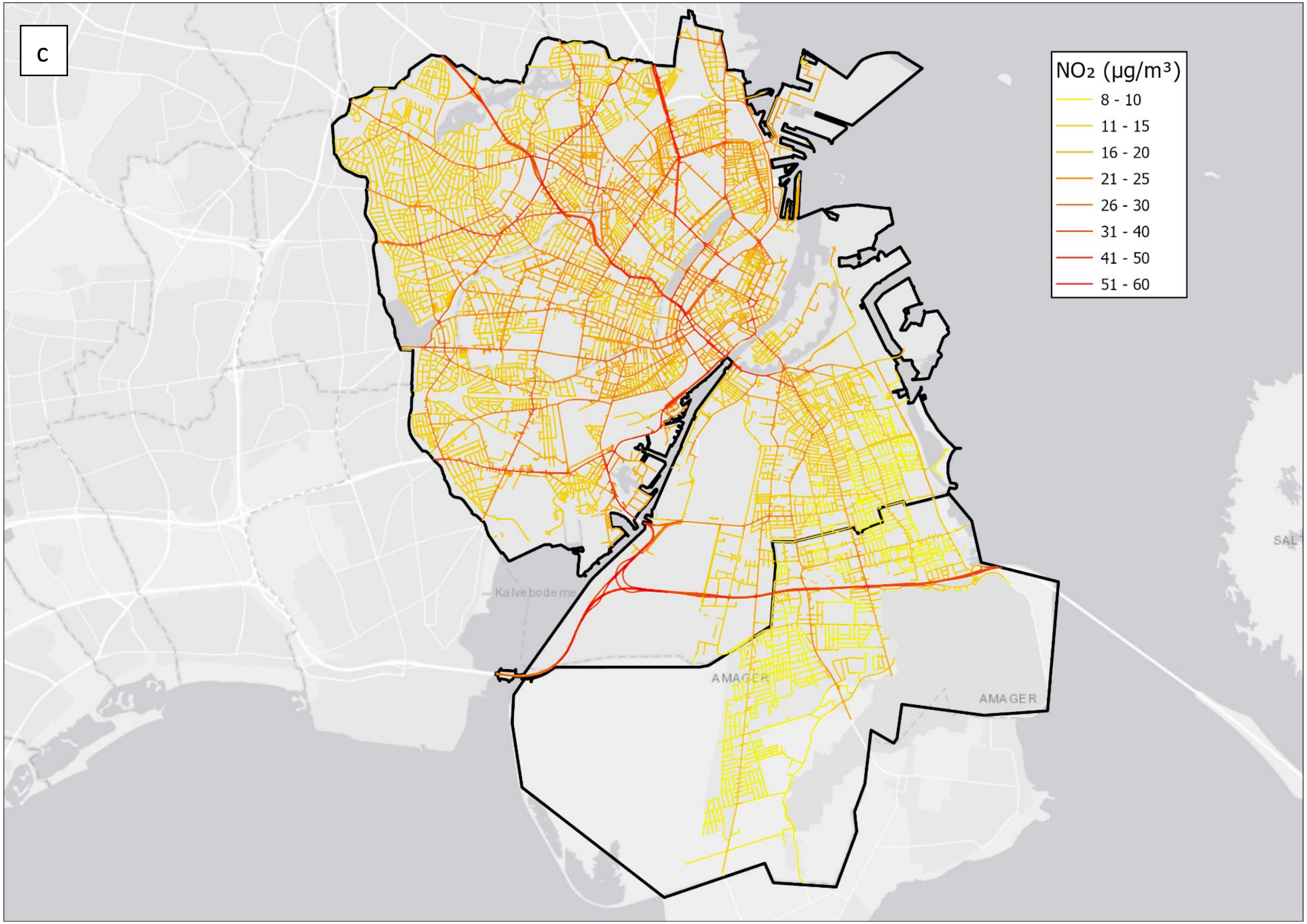
c
NO₂ (µg/m³)
8 - 10
11 - 15
16 - 20
21 - 25
26 - 30
31 - 40
41 - 50
51 - 60
Kalveboderne
AMAGER
AMAGER

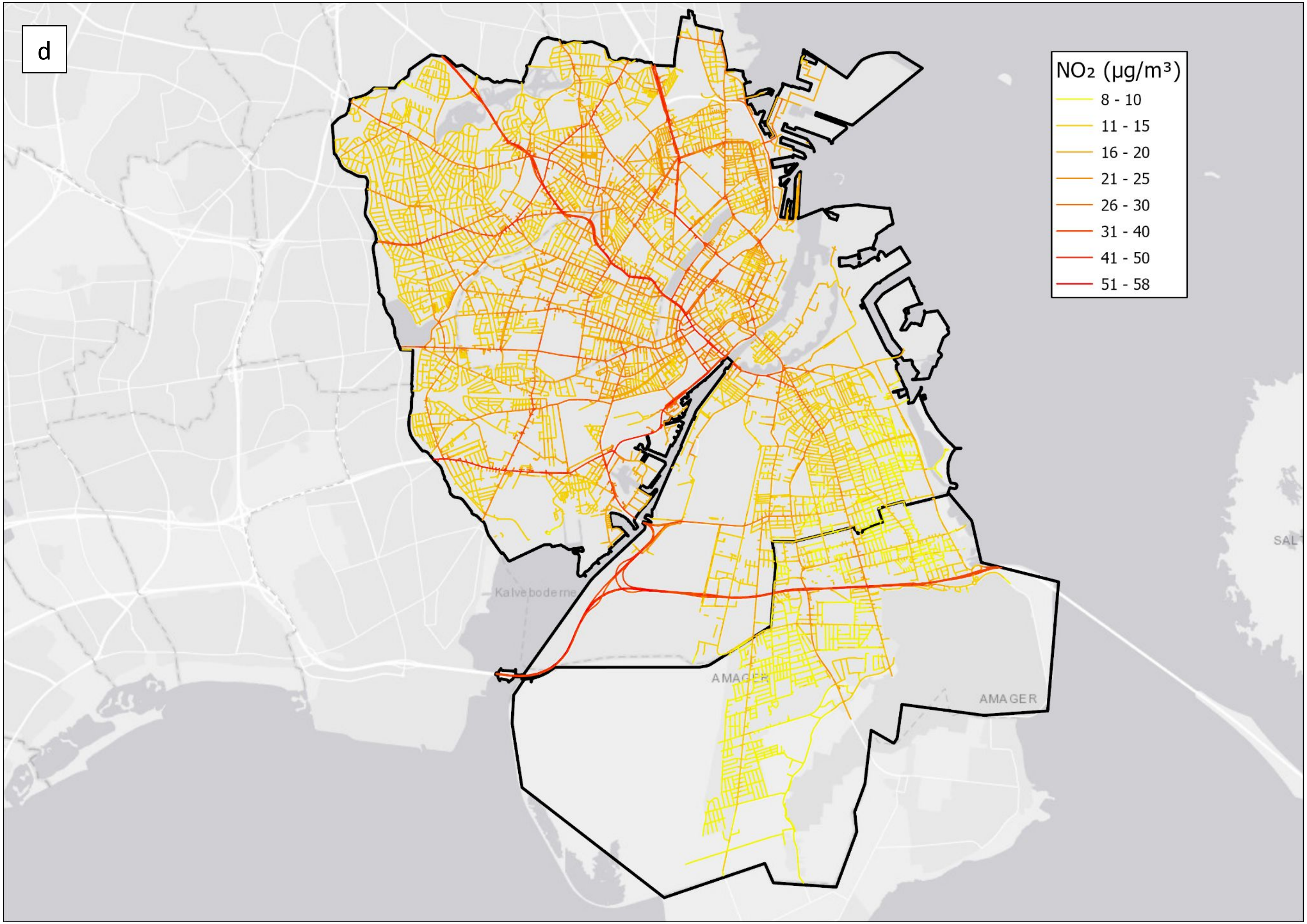
d
NO2 (µg/m³)
8 - 10
11 - 15
16 - 20
21 - 25
26 - 30
31 - 40
41 - 50
51 - 58
Kalvebøderne
AMAGER
AMAGER

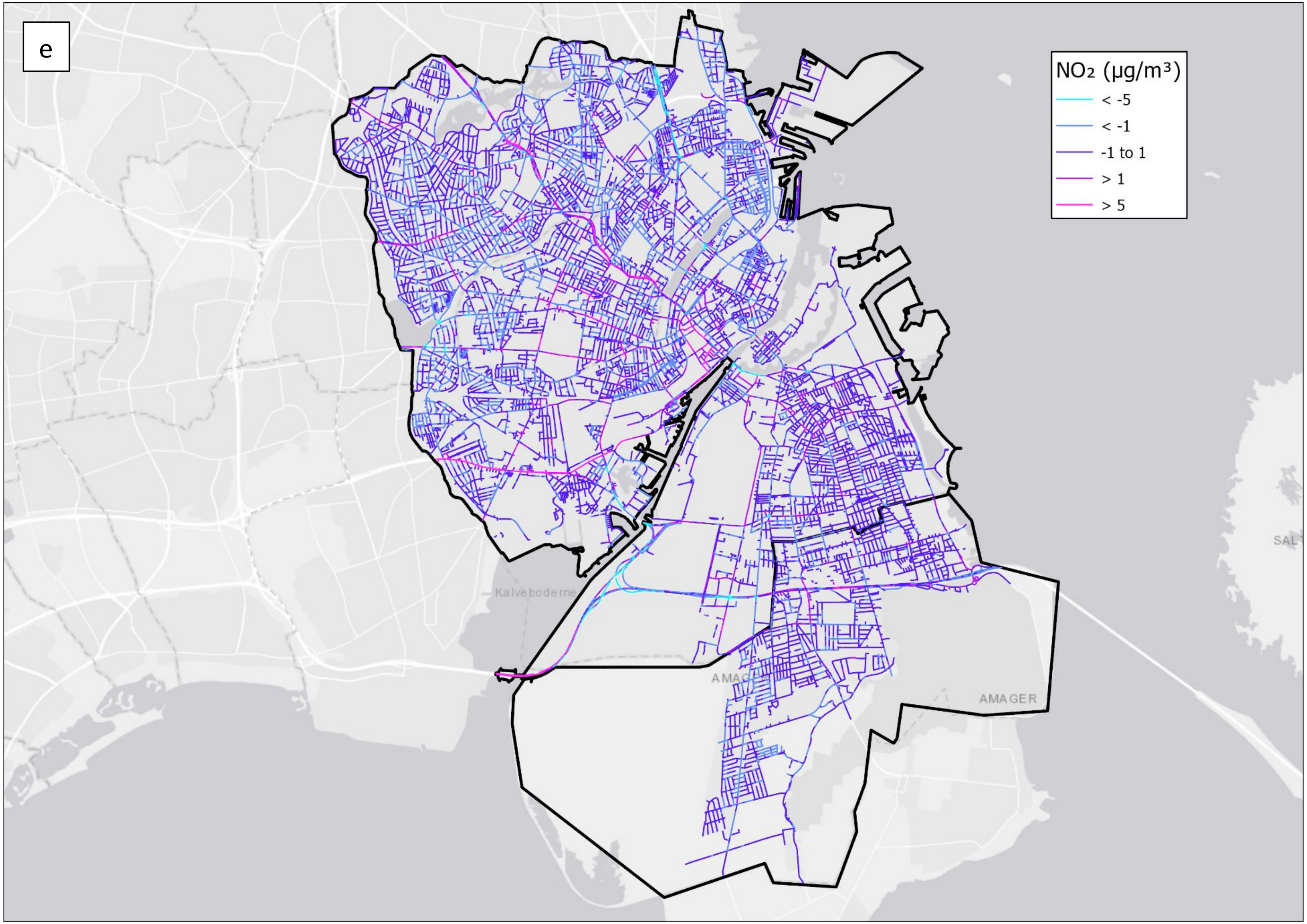

139

Figure B2: Distribution of measurements and predictions from the CAV and measurements for the external datasets (top). Subsequent boxplots represent the distributions with an equal number of sites between the CAV and external comparison dataset.

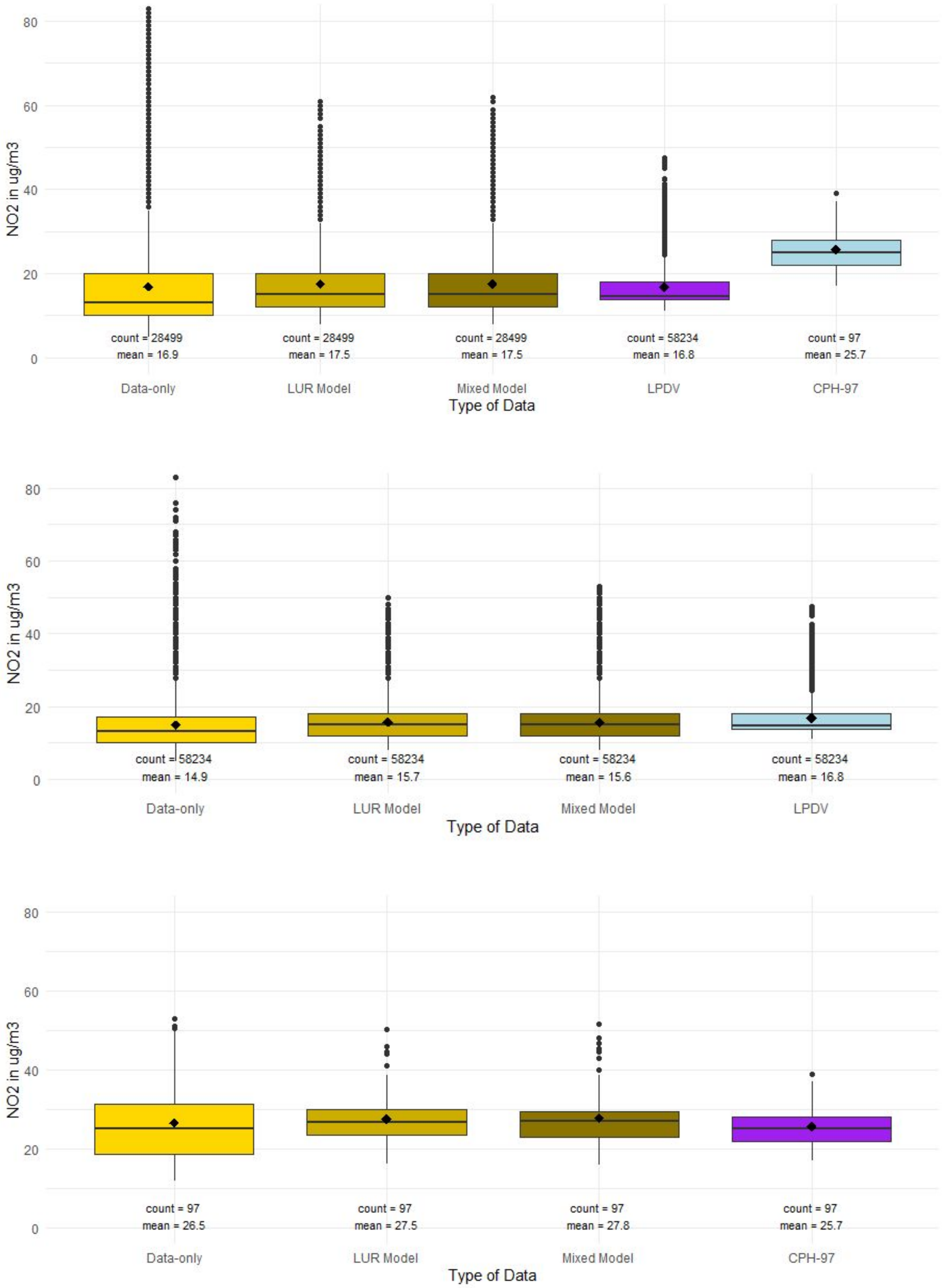

Figure B3: Scatterplot of CAV concentrations nearby routine measurement stations.

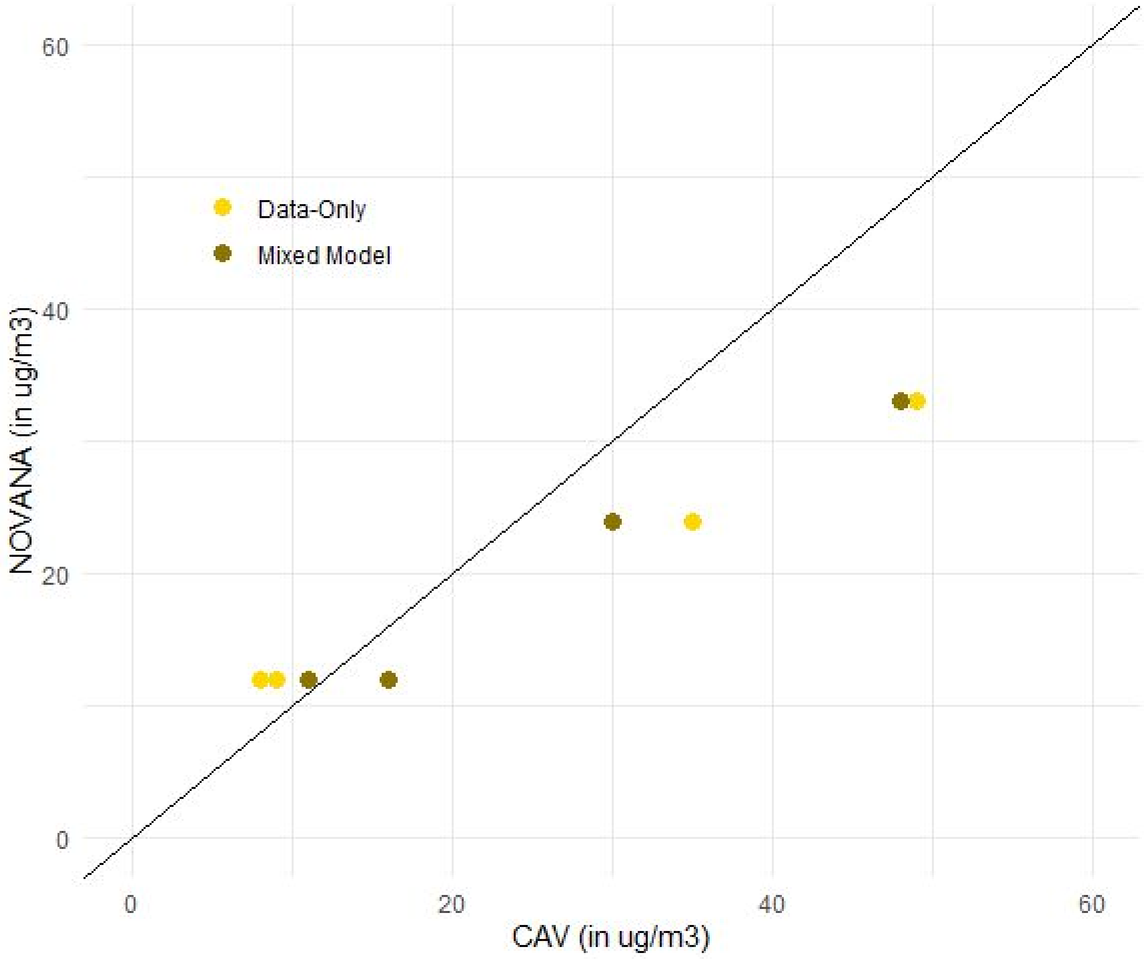

Figure B4: Scatterplots and Bland-Altman plots for comparisons between CAV and a) LPDV (n=58,234), b) CPH-97 predictions (n=97).

a)

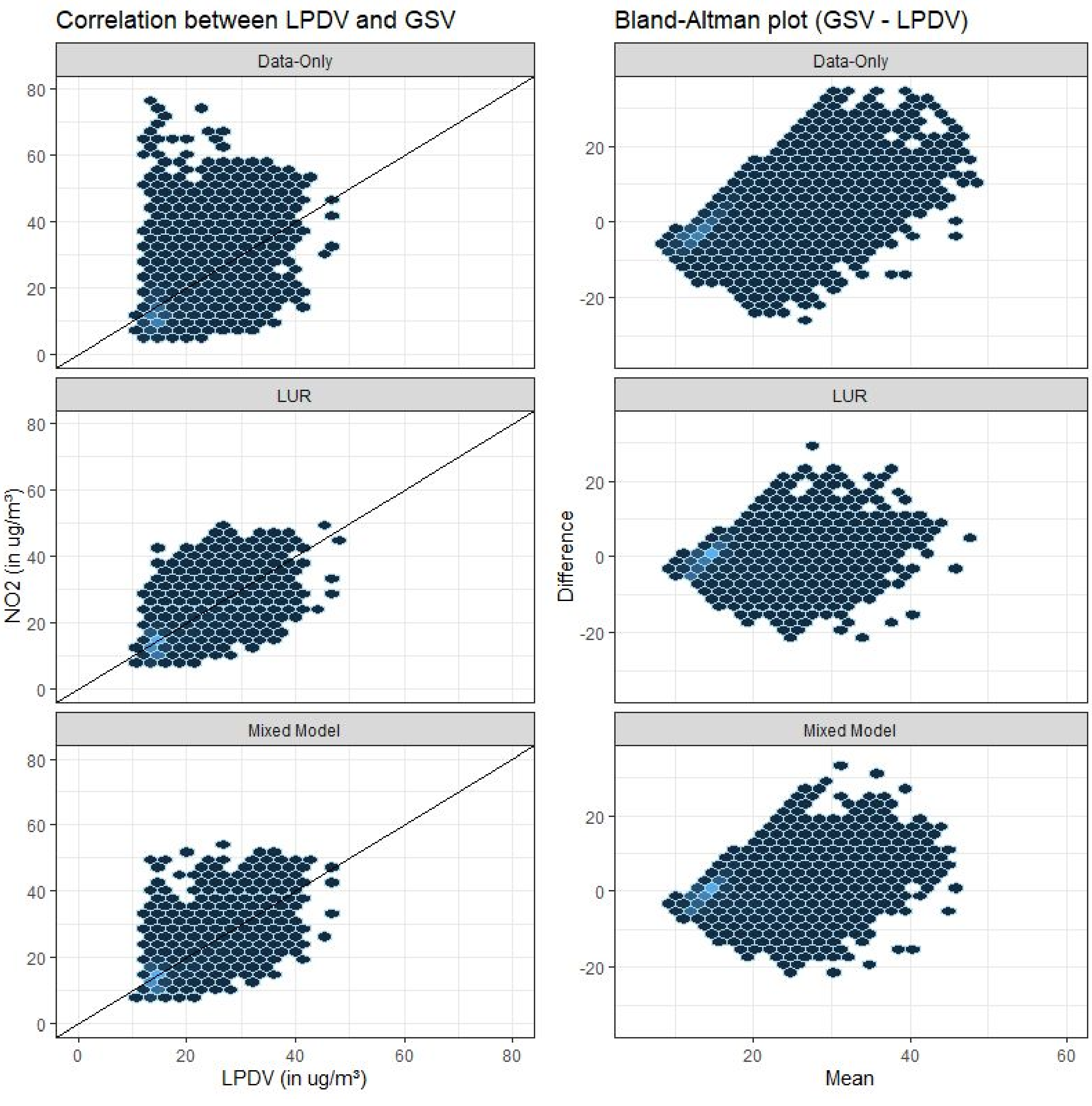

b)

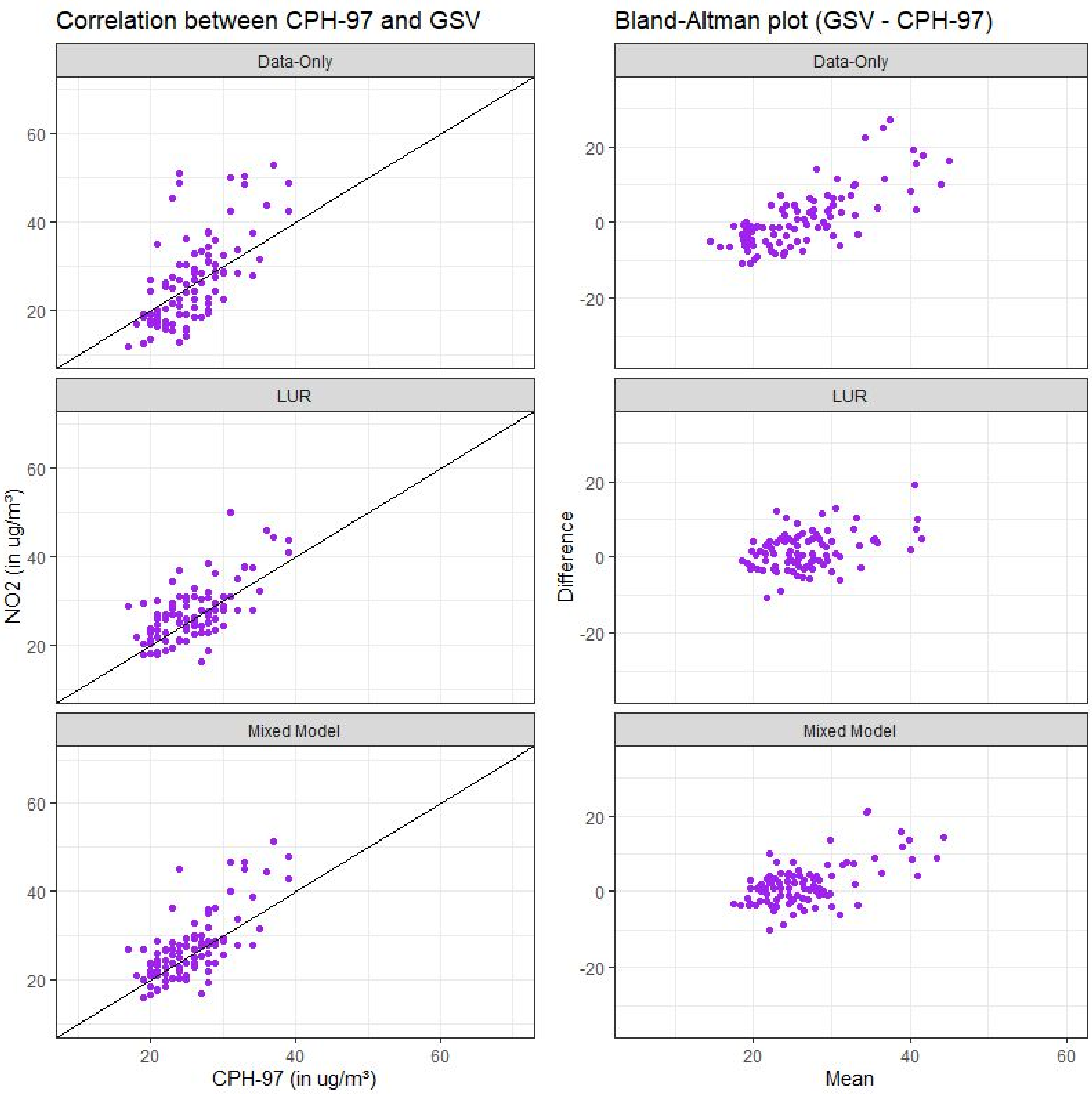
Correlation between CPH-97 and GSV
Bland-Altman plot (GSV - CPH-97)
Data-Only
LUR
Mixed Model
NO2 (in ug/m³)
CPH-97 (in ug/m³)
Difference
Mean
20
40
60
-20
0

References

(1) Kerckhoffs, J.; Hoek, G.; Messier, K. P.; Brunekreef, B.; Meliefste, K.; Klompmaker, J. O.; Vermeulen, R. Comparison of Ultrafine Particles and Black Carbon Concentration Predictions from a Mobile and Short-Term Stationary Land-Use Regression Model. *Environ. Sci. Technol.* **2016**, *50* (23), 12894–12902. https://doi.org/10.1021/acs.est.6b03476.

(2) Kerckhoffs, J.; Hoek, G.; Vlaanderen, J.; van Nunen, E.; Messier, K.; Brunekreef, B.; Gulliver, J.; Vermeulen, R. Robustness of Intra Urban Land-Use Regression Models for Ultrafine Particles and Black Carbon Based on Mobile Monitoring. *Environ. Res.* **2017**, *159*, 500–508. https://doi.org/10.1016/j.envres.2017.08.040.

(3) RIVM-LML. Luchtmeetnet.nl https://www.luchtmeetnet.nl/ (accessed Jun 11, 2021).

(4) Ellermann, T.; Nordstrøm, C.; Brandt, J.; Christensen, J.; Ketzel, M.; Massling, A.; Bossi, R.; Marie, L.; Camilla, F.; Steen, G.; Jensen, S.; Nielsen, O.-K.; Winther, M.; Poulsen, M. B.; Nygaard, J.; Klenø Nøjgaard, J. Videnskabelig Rapport Fra DCE-Nationalt Center for Miljø Og Energi. **2021**.

(5) Klompmaker, J. O.; Montagne, D. R.; Meliefste, K.; Hoek, G.; Brunekreef, B. Spatial Variation of Ultrafine Particles and Black Carbon in Two Cities: Results from a Short-Term Measurement Campaign. *Sci. Total Environ.* **2015**, *508*, 266–275. https://doi.org/10.1016/j.scitotenv.2014.11.088.

(6) Erceg-Hurn, D. M.; Mirosevich, V. M. Modern Robust Statistical Methods: An Easy Way to Maximize the Accuracy and Power of Your Research. *Am. Psychol.* **2008**, *63* (7), 591–601. https://doi.org/10.1037/0003-066X.63.7.591.

(7) NWB. Home :: Nationaal Wegenbestand https://nationaalwegenbestand.nl/ (accessed Apr 29, 2021).

(8) Solvang Jensen, S.; Hilling, K. GIS-Based National Road and Traffic Database 1960-2020. **2019**.

(9) EEA. Copernicus Land Monitoring Service - Corine Land Cover — European Environment Agency https://www.eea.europa.eu/data-and-maps/data/copernicus-land-monitoring-service-corine (accessed Apr 29, 2021).

(10) PBL. PBL Netherlands Environmental Assessment Agency | https://www.pbl.nl/en (accessed Apr 29, 2021).

(11) Khan, J.; Kakosimos, K.; Raaschou-Nielsen, O.; Brandt, J.; Jensen, S. S.; Ellermann, T.; Ketzel, M. Development and Performance Evaluation of New AirGIS – A GIS Based Air Pollution and Human Exposure Modelling System. *Atmos. Environ.* **2019**, *198*, 102–121. https://doi.org/10.1016/j.atmosenv.2018.10.036.
